# Allee Effect and Oncolytic Virotherapy: Different Therapeutic Outcomes for Different Tumour Growth

**DOI:** 10.1007/s11538-026-01746-9

**Published:** 2026-09-02

**Authors:** Jiawen Chen, Federico Frascoli, Tonghua Zhang

**Affiliations:** Department of Mathematics, Swinburne University of Technology, John Street, Hawthorn, VIC 3122 Australia

**Keywords:** Tumour, Oncolytic virotherapy, Allee effect, Tumour growth, Therapeutic outcomes

## Abstract

Tumour recurrence after oncolytic virotherapy is a critical clinical challenge, as solid cancers tend to persist and spread after such therapies alone. Often, mathematical models consider tumour growth without taking possible cooperative cell behaviours into account. Instead, the well-known Allee effect has the ability to drive low-density tumour dynamics and affect therapeutic outcomes. For this reason, we here explore the contribution of Allee effects into two of the most common cancer growth paradigms, e.g. the logistic and the Gompertz frameworks. Our analysis reveals that density-dependent cooperation fundamentally alters virotherapy efficacy. Weak effects can enable pseudo-extinction states where tumour populations collapse to undetectable levels, whilst strong effects can interestingly create non trivial, bistable outcomes. Initial tumour burden seems to be determinant: bifurcation analysis shows scenarios where modest increase in infection or decrease in clearance rates can shift outcomes dramatically. Notably, the Gompertz model exhibits multistability under marginal Allee thresholds, pointing at a possible explanation for spontaneous remission that have been clinically observed. Overall, these findings suggest that Allee effects may be an important factor in the future to adjust dosing schedules, reduce viral loads, lower recurrence risk and shorten therapeutic times. Our framework may also offer quantitative guidance for patient-specific regimes for virotherapy and combination therapies.

## Introduction

Cancer remains a leading cause of mortality worldwide, with treatment failure predominantly driven by recurrence and therapeutic resistance. Surgery, chemotherapy and radiotherapy are established techniques employed against tumours, whereas oncolytic virotherapy (OVT), although the effects of infections on tumours have been known for more than a century, still has not fully translated from trials to clinics. OVT emerged as a possible clinical approach a few decades ago, with the rise in genetical engineering and the ability of creating viruses that selectively infect and destroy cancer cells (Ribas et al. 2017; Zhong et al. 2025). Recently, its ability to also elicit targeted immune responses, co-adjuvate immunotherapy and combat immune evasion have emerged, with some encouraging yet incomplete results in preclinical and murine models (Chen et al. 2024; Nguyen et al. 2022b; Yun et al. 2022; Jhawar et al. 2023; Yang et al. 2025; Xuan et al. 2025). Despite decades of promises, achieving complete remission in most common types of tumours remains a fundamental challenge, especially if OVT is used in isolation, e.g., without the help of other techniques.

Mathematical modeling has been very helpful in characterising the dynamics and the typical outcomes of tumours under different OVTs. For example, deterministic models reveal dose-response thresholds (Wodarz 2001; Komarova and Wodarz 2010; Guo and Dobrovolny 2023; Ramaj and Zou 2023) and stochastic frameworks show the effect of tumour heterogeneity on viral extinction (Yuan and Allen 2011; Rajalakshmi and Ghosh 2018; Camara et al. 2022; Morselli et al. 2026a). Spatial models explained oscillatory patterns in heterogeneous microenvironments (Bhatt et al. 2022; Morselli et al. 2023; Baabdulla and Hillen 2024; Morselli et al. 2026b). However, standard logistic and Gompertz growth laws are density-dependent, but they do not explicitly incorporate positive density dependence or cooperative low-density effects at low tumour densities. Consequently, such formulations may fail to capture critical low-density phenomena observed clinically, where microscopic tumour clusters exhibit non-monotonic growth dynamics distinct from established lesions.

Notably, recent evidence reveals that tumour growth at low densities exhibits cooperative dynamics. For example, it is known that sub-threshold tumour clusters fail to sustain proliferation due to insufficient nutrient pooling and compromised intercellular signalling (Guzelsoy et al. 2025) and that low-density populations also display epigenetic instability and antiviral pathways (Shen et al. 2021; Endicott et al. 2022; You et al. 2024). Another important driver of collective responses are mutations: cancer stem cell subpopulations accumulate mutations during dormancy, which are driving elements for recurrence (Sewalt et al. 2016; Cañellas-Socias et al. 2022). These observations are coherent with the typical phenomenology of the Allee effect, wherein per-capita growth rates decline below a critical density threshold. The effect traces back to early aggregation experiments by  Allee and Bowen (1932), who reported mass protection against colloidal silver in goldfish. Originally described for social species (Stephens and Sutherland 1999), Allee dynamics arise when cooperative behaviours such as nutrient sharing, paracrine signalling, or collective immune evasion become ineffective at low densities (Courchamp et al. 2008). There seems to be increasingly compelling biological evidence within cancer, including cooperative nutrient scavenging, secretion of autocrine growth factors and the formation of protective niches that shield small cell clusters from immune attack (Sewalt et al. 2016; Peng and Zhang 2016; Liu et al. 2019; Yu et al. 2018; Xu et al. 2019; Liu et al. 2020; Shen et al. 2021; Endicott et al. 2022; Cañellas-Socias et al. 2022; You et al. 2024; Guzelsoy et al. 2025).

Our study concentrates on three questions that we believe are central to optimising virotherapy in the presence of behaviours in line with Allee or Allee-type effects. Firstly, we are interested in how size thresholds alter the balance between tumour eradication and persistent viral infections that do not sufficiently reduce the tumour load. Secondly, we want to explore which viral characteristics enable spontaneous clearance if viral loads are reduced. Finally, it is therapeutically valid to consider if there exist situations where modest dose adjustments can produce significant gains and prolonged benefits to patients. The rest of the paper is structured as follows. After we describe and justify our choices of models, we provide a comprehensive stability analysis and bifurcation diagrams of relevant parameter dependence for the models (Sect. 3). We also validate predictions through numerical simulations calibrated to clinical data (Sect. 4) and finally discuss implications for adaptive treatment protocols (Sect. 5). Our findings reveal that Gompertz dynamics exhibit richer multistability under weak Allee effects, whilst logistic dynamics display simpler threshold-driven transitions. Both frameworks demonstrate that strategic exploitation of density-dependent growth could substantially improve therapeutic outcomes, based on tumours’ growth characteristics, while reducing toxicity and cost.

## Growth Models and Allee Effects

The Allee effect is usually categorised as strong, presenting with an extinction threshold, if a critical population threshold exists below which the population collapses. Conversely, it is considered as weak, and shows reduced growth without a threshold (Wei et al. 2020), if the growth rate is merely suppressed at low densities but remains positive (Deredec and Courchamp 2003; Zu and Mimura 2010; Xue and Liu 2015). This density-dependent regulation is useful because it provides a simple, phenomenological, mechanistic basis for discriminating between dormant, quiescent tumours and growing tumours that progress to overt disease (Böttger et al. 2015; Konstorum et al. 2016; Sardar and Khajanchi 2022). Furthermore, recent mathematical modelling has found that the Allee effect can capture density-dependent growth patterns observed in colorectal cancer progression data, highlighting the potential relevance of such mechanisms for therapeutic decision-making (Delitala and Ferraro 2020). For a strong Allee effect, the growth equation that is most often used and that involves a logistic growth term is given by

$$ \frac{dN}{dt} = rN\left( 1 - \frac{N}{K}\right) \left( \frac{N}{K} - \frac{A}{K}\right) , $$

where *N* denotes the number of tumour cells, *A* the so-called critical threshold and *K* is the microenvironmental carrying capacity (Lewis and Kareiva 1993; Arancibia-Ibarra 2019). To integrate that dynamics into oncolytic virotherapy, we use the Gompertz-based framework of Jenner et al. (Kim et al. 2011; Jenner et al. 2018, 2019) and introduce a density-dependent correction. We evaluate various expressions for the Allee term, introducing a modulating factor $\Omega (U,\theta )$ to alter the baseline Gompertz growth rate $\bar{g}(U) = \bar{m} \ln (K/U)$ (Courchamp et al. 2008). Using the following term:

$$ g(U) = \bar{g}(U) \cdot \Omega (U,\theta ) = \bar{m} \ln \left( \frac{K}{U}\right) \Omega (U,\theta ), $$

where $\Omega (U,\theta )$ can have different functional forms, various interesting growth cases can be considered. In Appendix A, a comparative analysis of a number of models is presented, with technical details and derivations. Not all cases are biologically sound and here we focus solely on the two formulations that we deem the most realistic, since they are in line with experimental data and clinical practice.

In what follows, following Jenner et al. (2018, 2019), we write both systems (1)–(2) in reduced nondimensional form. Tumour-cell densities are normalised by $\bar{U}=10^6$ cells $\hbox {mm}^{-3}$, so that U=1 corresponds to $10^6$ cells $\hbox {mm}^{-3}$. The same cell-density scaling is used for *I*, *K*, and θ. The original free-virus variable is divided by the viral burst-size parameter, placing *V* on a cell-equivalent scale, and the infection-rate coefficient is absorbed into the time scale. With these scalings, all terms in (1)–(2) have units of scaled cell density per reduced time (see Table 1). Let us now introduce the first model. The equations are given as follows and we choose to label it the “Unified Allee Gompertz model” for reasons explained shortly:

1 $$\begin{aligned} {\left\{ \begin{array}{ll} \displaystyle \frac{dU}{dt} = mU(U-\theta ) \ln \left( \frac{K}{U}\right) - \frac{UV}{U+I}, \\ \displaystyle \frac{dI}{dt} = \frac{UV}{U+I} - \xi I, \\ \displaystyle \frac{dV}{dt} = \xi I - \gamma V, \end{array}\right. } \end{aligned}$$

Here, *U*(*t*) denotes the density of uninfected tumour cells, *I*(*t*) the density of infected tumour cells, and *V*(*t*) the rescaled free-virus density. Parameter definitions are given in Table 1. Here, we take $m=\bar{m}/{K}$, $\Omega (U, \theta )=\frac{U-\theta }{K}$ with $-K \le \theta \ll K$, meaning that Ω<0 for U<θ (negative growth), Ω=0 at U=θ (threshold) and $\Omega \in (0,2)$ for θ<U<K (bounded positive growth). This model provides efficiency in cooperation that inversely scales with carrying capacity: in environments where the number of cells is sufficiently large (i.e., *K* large), the benefits of cooperation are reduced. Conversely, confined spaces (i.e., *K* small) are described as promoting proximity among cells, which in turn enhances cooperative interactions (Jenner et al. 2018, 2019).

**Table 1:** Model parameters and their biological interpretation in the reduced nondimensional OVT systems

Description	Parameter	Baseline value	References
Microenvironmental carrying capacity	*K*	100	(Kim et al. 2011; Böttger et al. 2015) (Jenner et al. 2018; Delitala and Ferraro 2020)
Allee threshold (tumour viability limit)	θ	${-}{-}$	(Kim et al. 2011; Böttger et al. 2015) (Jenner et al. 2018; Delitala and Ferraro 2020)
Tumour cell Gompertz growth rate in Gompertz	*m*	0.01	(Kim et al. 2011; Böttger et al. 2015) (Jenner et al. 2018; Delitala and Ferraro 2020)
Tumour cell Logistic growth rate	*a*	0.0001	(Kim et al. 2011; Böttger et al. 2015) (Jenner et al. 2018; Delitala and Ferraro 2020)
Reduced infected-cell lysis rate	ξ,b	0.01	(Kim et al. 2011; Böttger et al. 2015) (Jenner et al. 2018)
Extracellular viral clearance rate	γ,c	0.06	(Kim et al. 2011; Böttger et al. 2015) (Jenner et al. 2018)

All variables and parameters are dimensionless after the cell-density, burst-size and time rescalings described in the text

This model is endowed with a number of advantages over other, alternative functional forms. Firstly, it is bounded at high density: contrary to other models discussed in the Appendix (for instance (5) or (8)) here the Allee correction saturates near *K*, preserving the typical slowing down of Gompertz growth close to carrying capacity. Secondly, it represents a unified framework, hence its name, where a single parameter θ can govern both strong (θ>0) and weak (θ<0) behaviours. It also preserves smoothness for the dynamics near extinction: unlike model (5), for instance, with logarithmic singularity, here (U-θ)/K produces finite negative growth for U<θ. Finally, a direct relationship between the Allee threshold and environmental carrying capacity is supported by evidence that cooperative mechanisms such as autocrine signalling and metabolite exchange impose density-dependent constraints on tumour growth, effectively tying minimal viable density to resource and microenvironmental limits (Gerlee et al. 2022; Hershey et al. 2023), this view is further reinforced by models linking density dependence to post-resection recurrence (Neufeld et al. 2017). Overall, this model has the best balance between simplicity and biological realism, and is able to represent both strong and weak Allee regimes within a unified framework. For all these reasons, it is chosen as the first system in our analysis.

Gompertz dynamics is also not the only way of describing growing trajectories of developing cancers. It effectively captures late-stage slow down of tumours (Gerlee 2013; Vaghi et al. 2020), but often exhibits a too fast initial growth rate, which can be biologically implausible for microscopic lesions or slowly growing cellular aggregates (Benzekry et al. 2014). It is therefore valuable for our approach to use an analogous logistic-type framework, that, in line with the previous observations, we label as a “Unified Allee Logistic Model”:

2 $$\begin{aligned} {\left\{ \begin{array}{ll} \displaystyle \frac{dU}{dt} = aU(K-U)(U-\theta ) - \frac{UV}{U+I}, \\ \displaystyle \frac{dI}{dt} = \frac{UV}{U+I} - bI, \\ \displaystyle \frac{dV}{dt} = bI - cV, \end{array}\right. } \end{aligned}$$

where the incidence term UV/(U+I) is a frequency-dependent infection term, following Jenner et al. (2018, 2019)-type OVT models. Here U/(U+I) represents the fraction of uninfected tumour cells in the local tumour-cell population, so viral infection is normalised by total tumour burden rather than increasing as a mass-action term *UV*. The remaining transfer, production and clearance terms are taken to be linear, corresponding to first-order infected-cell lysis, virus production and extracellular viral decay in a reduced OVT model.

**Fig. 1:**
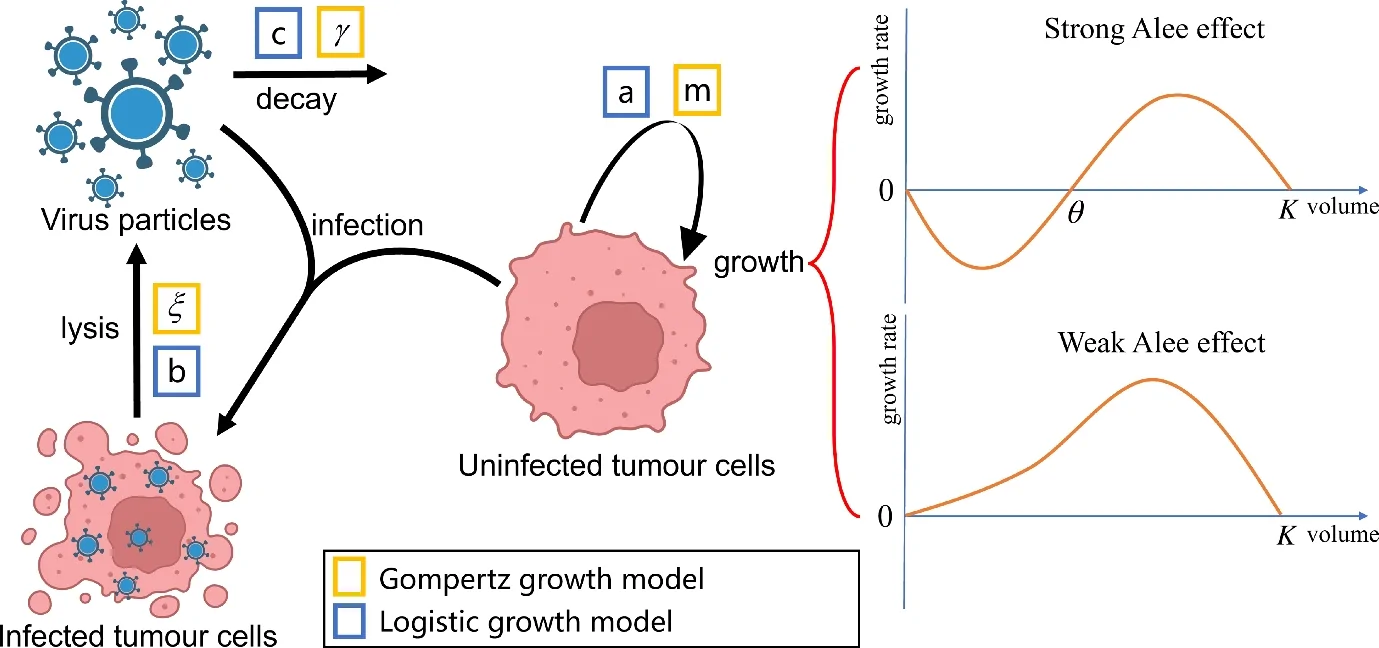
Schematic of tumour-virus interactions in oncolytic virotherapy models. Uninfected tumour cells *U*(*t*) grow according to either Gompertz (yellow parameters: *m*, ξ, γ) or logistic (blue parameters: *a*, *b*, *c*) laws modulated by Allee threshold θ. Infection converts *U* to *I*, which lyse to release virus *V*. Both models share similar infection-lysis-clearance dynamics but differ in intrinsic growth dynamics and parameter sensitivity (Color figure online)

The logistic formulation provides an often more realistic early-phase growth but frequently underestimates late-stage inhibition and volume effects that limit tumour proliferation (Vaghi et al. 2020; Voulgarelis et al. 2022). Given the characteristic of the logistic model and its popularity in the literature, a comparison with the Gompertz case (Fig. 1) is thus carried out, enabling integration of complementary strengths and discussion of intrinsic shortcomings for both models. The functional forms that we discard, as mentioned, are highlighted in the Appendix A.

## Stability Analysis for Both Models

### Gompertz Model: Equilibrium and Stability

We first summarise the equilibrium and local stability structure of the Gompertz model  (1). Let us now characterise the equilibrium structure of model (1) and establish conditions for local stability. The analysis reveals how Allee thresholds partition parameter space into distinct therapeutic regimes. Model (1) admits multiple steady states depending on the threshold θ and viral parameters ξ, γ. We identify four biologically relevant equilibria:

**Biologically Relevant Isolated State (BRIS):** $\overline{E}_0(0,0,0)$ represents complete tumour and viral clearance. While mathematically singular due to the denominator U+I in the infection term, this state corresponds to successful therapy where all populations fall below detection limits (clinically, $U < 10^{-6}$ tumour cells per $mm^3$). Its biological attractiveness under strong Allee effects will be demonstrated numerically shortly.

**Boundary equilibria:** $\overline{E}_1(K,0,0)$ and $\overline{E}_2(\theta ,0,0)$ represent viral-free states where uninfected cells reach carrying capacity or threshold density, respectively. Equilibrium $\overline{E}_2$ exists only when $0 \le \theta < K$ (strong Allee regime).

**Coexistence equilibria:** $$\overline{E}^*$$, $$\overline{E}^{**}$$, $$\overline{E}^{***}$$ describe persistent infection states where tumour and virus coexist (see Table 2). At any such equilibrium $$(U^*, I^*, V^*)$$, infected cells and free virus scale with uninfected cells according to $$I^* = [(1-\gamma )/\gamma ]U^*$$ and $$V^* = [\xi (1-\gamma )/\gamma ^2]U^*$$, requiring γ<1 for positivity. Let $$\Lambda = \dfrac{\xi (1-\gamma )}{\gamma }$$ for convenience, the density $$U^*$$ satisfies:

3 $$\begin{aligned} m(U^*-\theta ) \ln \left( \frac{K}{U^*}\right) = \frac{\xi (1-\gamma )}{\gamma }=\Lambda . \end{aligned}$$

Defining $f(x) = m(x-\theta )\ln (K/x)$, the number of solutions $U^*$ to (3) depends on the shape of *f*. For weak Allee effects (θ<0), the critical points of *f*(*x*) are obtained by solving $f'(x)= m[\ln (K/x) - 1 + \theta /x]=0$, which can be rearranged into a transcendental equation of the form $z e^{z} = {\theta e}/{K}$, whose solutions are expressed in terms of the Lambert *W* function (Corless et al. 1996). For -1/e<θe/K<0, two real branches exist, namely $W_{-1}$ and $W_{0}$, similarly to other models where interactions between tumours and immune responses have been considered (Frascoli et al. 2014). Hence, the two critical points are given by $x_1 = \dfrac{\theta }{W_{-1}\left( \dfrac{\theta e}{K} \right) }, x_2 = \dfrac{\theta }{W_0\left( \dfrac{\theta e}{K} \right) },$ with $0<x_1 < x_2$. These points determine the local extrema of *f*(*x*).

Now, let us define $$\overline{U}^*> \overline{U}^{**}> \overline{U}^{***}$$. Detailed analysis (see Appendix B.1 for a thorough discussion) establishes the following:

(i) If $-K \le \theta \le -Ke^{-2}$: a unique equilibrium $\overline{E}^*$ exists.
(ii) If $$-Ke^{-2}< \theta < 0$$: one or three equilibria are present, depending on whether Λ falls within the range $$(f(x_1), f(x_2))$$, where $$ x_1 = \dfrac{\theta }{W_{-1}\left( \dfrac{\theta e}{K} \right) }$$ and $$x_2 = \dfrac{\theta }{W_0\left( \dfrac{\theta e}{K} \right) } $$ are the roots of $f^{\prime }\left(x\right)=0$.
(iii) If $0 \le \theta < K$: two equilibria are found when $\Lambda < f(x_2)$, where $x_2=\dfrac{\theta }{W_0 \left( \dfrac{\theta e}{K}\right) }$. Here $\theta e/K\ge 0$, hence the real-valued Lambert *W* is uniquely given by the principal branch $W_0$ (see Appendix B.1 for details).

**Fig. 2:**
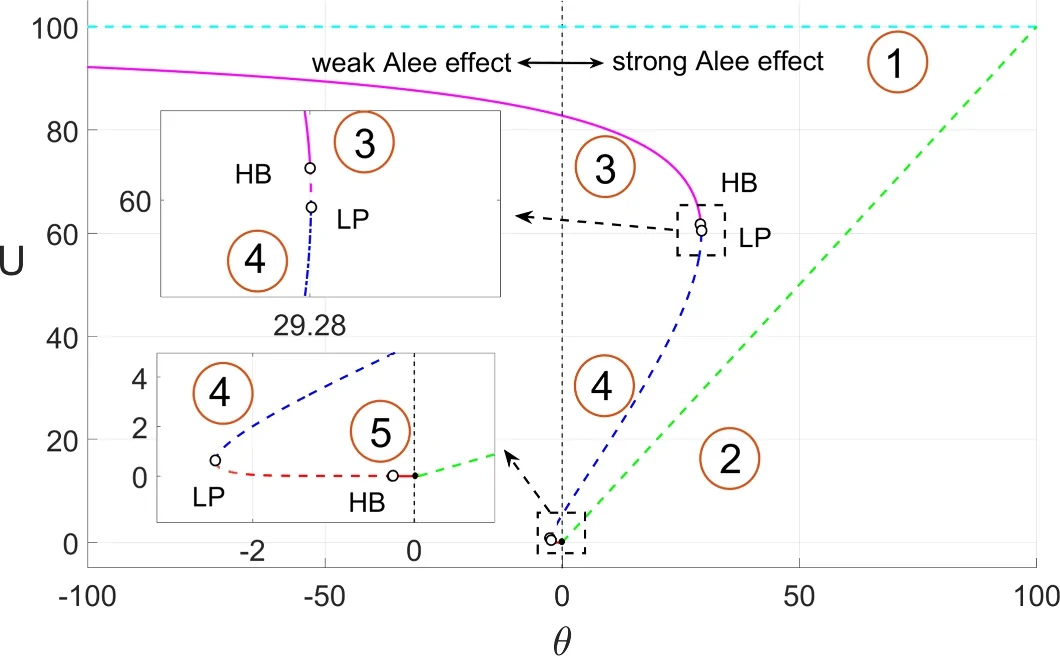
Equilibrium structure and stability landscape in the model with Gompertzian growth (Eq. 1). Solid curves denote stable states; dashed curves indicate unstable ones. The vertical line at θ=0 separates the weak Allee (left, θ<0) from the strong one (right, θ>0). Labels are as follows: (1, cyan) tumour-only state $$\overline{E}_1$$; (2, green) threshold state $$\overline{E}_2$$; (3, magenta) primary coexistence $$\overline{E}^*$$; (4, blue) unstable coexistence $$\overline{E}^{**}$$; (5, red) coexistence $$\overline{E}^{***}$$. “HB” indicates a Hopf bifurcation, “LP” denotes a saddle-node (noted as a "limit point" by the software) bifurcation. Three equilibria coexist only for $$-Ke^{-2}< \theta < 0, $$ when viral parameters satisfy $$f(x_1)< \xi (1-\gamma )/\gamma < f(x_2)$$. This multistability gives rise to what we label as pseudo-extinction, a phenomenon absent in the logistic model (see following Figure). Parameters are given by: m=0.01, K=100, ξ=0.01, γ=0.06 (Color figure online)

Details of the above points are collated in Table 2, whereas Fig. 2 captures their loci as a function of θ. Notably, the branch point $\theta = -Ke^{-2}$ separates regimes with a single state from those with multiple coexisting states, which is a feature not observed in oncolytic models with ordinary Gompertz growth.

**Table 2:** Equilibrium existence conditions of Gompertz model (1)

Equilibrium	Coordinates	Existence Condition
$\overline{E}_1$	(*K*, 0, 0)	Always
$\overline{E}_2$	(θ,0,0)	0<θ<K
$\overline{E}^*$	$(U^*, I^*, V^*)$	γ<1 and $-K \le \theta <0$, or γ<1, $0 \le \theta <K$, $\Lambda \le f(x_2)$
$$\overline{E}^{**}$$	$$(U^{**}, I^{**}, V^{**})$$	γ<1, $$-Ke^{-2}<\theta <0$$, $$f(x_1)\le \Lambda \le f(x_2)$$, or γ<1, $$0 \le \theta <K$$, $$\Lambda \le f(x_2)$$
$$\overline{E}^{***}$$	$$(U^{***}, I^{***}, V^{***})$$	γ<1, $$-Ke^{-2}<\theta <0$$, $$ f(x_1)\le \Lambda \le f(x_2)$$

The value of threshold $\theta =-Ke^{-2}$ demarcates regions with differing numbers of states and $\Lambda =\xi (1-\gamma )/\gamma$

Local stability is determined by the Jacobian matrix at each equilibrium, and the characteristic polynomial with detailed derivations is left for Appendix B.2. Key results can be summarised as follows:

#### Theorem 1

(Boundary equilibria of the Gompertz model (1)) For boundary equilibria:eumerate

(i) If γ>1, then $\overline{E}_1(K,0,0)$ is locally asymptotically stable for all θ. This reflects rapid viral clearance preventing sustained infection.
(ii) Equilibrium $\overline{E}_2(\theta ,0,0)$ is always unstable when it exists (θ>0).

Biologically, the meaning of Theorem 1(i) is that high viral clearance rates (γ>1, corresponding to viral half-lives under one day) make oncolytic therapy ineffective regardless of initial conditions. The tumour inevitably escapes to carrying capacity. Conversely, the unstable character of $\overline{E}_2$ confirms that populations at the Allee threshold are inherently unstable, either collapsing to extinction or expanding beyond threshold.

For coexistence equilibria, stability depends on parameter $h = m(U^* - \theta ) - mU^* \ln (K/U^*)$, which is, in a way, a measure of the tumour’s intrinsic ability to regulate its growth. Mechanistically, $h = -U^* f'(U^*)$ measures a negative feedback intensity: larger *h* implies stronger density-dependent suppression. The value of $z_2$, whose quite involved expression is explicitly derived in Appendix B, represents the destabilising potential of the virus-cell feedback loop, determined solely by viral parameters ξ and γ. So, condition $h > z_2$ thus requires that tumour self-regulation dominates over the virally induced oscillatory forcing: Fig. 2 helps visualising this stability landscape. Noticeably, it is also revealed that a Hopf bifurcation occurs near $\theta \approx 29.28$ (parameters: m=0.01, ξ=0.01, γ=0.06), demarcating stable coexistence from oscillatory collapse, as we will shortly show.

Another important aspect of the model is described by the following considerations:

#### Theorem 2

(Coexistence equilibria of the Gompertz model (1)) Assume 0<γ<1. *h* and $z_2$ be defined as in Appendix B.2, Eq. (14). Then:

(i) If $-K \le \theta < 0$, equilibrium $\overline{E}^*$ is locally asymptotically stable provided $h > z_2$.
(ii) When multiple equilibria exist ($$-Ke^{-2}< \theta < 0$$), $$\overline{E}^{**}$$ is always unstable, while $$\overline{E}^{***}$$ is stable if $$f(x_1)<\Lambda < f(x_2)$$ and $$h > z_2$$, where $$ x_1 = \dfrac{\theta }{W_{-1}\left( \dfrac{\theta e}{K} \right) }$$ and $$x_2 = \dfrac{\theta }{W_0\left( \dfrac{\theta e}{K} \right) } $$.
(iii) For strong Allee effects $(0 \le \theta < K)$, $\overline{E}^*$ is stable when $h > z_2$ and $\Lambda < f(x_2)$.

There is an important consequence for treatment that is elucidated by the stability analysis above. For tumours with weak self-regulation (small *h*), even moderate viral pressure can trigger oscillatory dynamics that may delay but ultimately enable clearance, as we will delve into in detail, in the next Sections. On the contrary, tumours with high *h* values require sustained, high viral loads to prevent stable coexistence. Parameter $z_2$ provides a patient-specific index: when $h < z_2$ higher doses are required, whilst for $h > z_2$ tumours can be given low-dose injections and still respond to therapy.

### Logistic Model: Equilibrium and Stability

We next state the corresponding analytical results for the logistic model (2), in parallel with the Gompertz results above. Although the two models share the same infection–lysis–clearance mechanism, their different growth laws lead to distinct equilibrium structures. The logistic model (2) shares a similar stability structure with the Gompertz model, but its different mode of growth gives rise to simpler bifurcation patterns. We here emphasise the key differences, with a detailed derivation in Appendix B.3.

Similarly to the previous case, model (2) admits BRIS $E_0(0,0,0)$, boundary equilibria $E_1(K,0,0)$ and $E_2(\theta ,0,0)$, with the latter only for 0<θ<K. Coexistence equilibria are determined by the following quadratic expression:

4 $$\begin{aligned} U^{2} - (K+\theta )U + K\theta + d = 0, \quad d = \frac{b(1-c)}{ac} > 0. \end{aligned}$$

Unlike the transcendental Eq. (3) for the Gompertz case, this quadratic admits closed-form solutions:

$$\begin{aligned} U_{1,2} = \frac{K+\theta \pm \sqrt{(K-\theta )^2 - 4d}}{2}. \end{aligned}$$

Defining $\theta _a = -d/K < 0$ and $\theta _b = K - 2\sqrt{d}$, we find the following cases:

- If $-K \le \theta \le \theta _a$, there is a unique equilibrium $E^*(U_1, I^*, V^*)$ with $U_1 > (K+\theta )/2$.
- If $$\theta _a< \theta < \theta _b$$, there are two equilibria $$E^*$$ and $$E^{**}$$ with $$U_1> (K+\theta )/2 > U_2$$.
- If $\theta = \theta _b$, there exist two identical equilibria $U_1 = U_2 = (K+\theta )/2$, corresponding to a saddle-node bifurcation.
- If $\theta \ge \theta _b$, there are no coexistence equilibria.

**Fig. 3:**
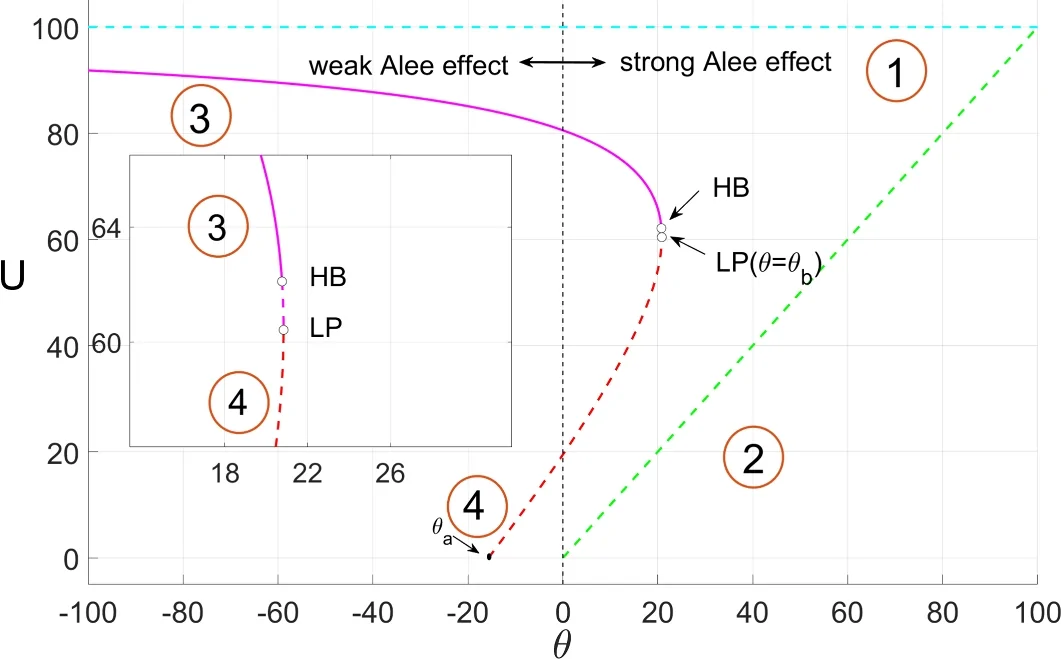
Schematic diagram of the existence and stability of equilibrium points in model (2) when c<1. Solid lines denote stable states, while dashed lines indicate instability. The diagram is bisected by a dashed line at θ=0, with the left section representing the weak Allee effect in tumour growth and the right section corresponding to the strong Allee effect. Labels are as follows: (1, cyan) tumour-only state $${E}_1$$; (2, green) threshold state $${E}_2$$; (3, magenta) primary coexistence $${E}^*$$; (4, red) unstable coexistence $${E}^{**}$$. “HB” denotes a Hopf bifurcation, and “LP” denotes a saddle-node (noted as a "limit point" by the software) bifurcation. The parameter values are taken as a=0.0001, K=100, b=0.01, c=0.06 (Color figure online)

Table 3 and Fig. 3 give details about the above structures. Notably, the model with logistic growth does not present a three-equilibria regime as it occurs for the model with Gompertzian growth. Its simpler structure reflects the polynomial nature of the growth terms and has clinically relevant consequences: fewer coexistence regimes and sharper therapeutic thresholds can change the approach to the design of effective treatment strategies.

**Table 3:** Equilibrium existence conditions of Logistic model (2)

Equilibrium	Coordinates	Conditions for existence
$E_1$	(*K*, 0, 0)	Always
$E_2$	(θ,0,0)	0<θ<K
$E^*$	$(U^*, I^*, V^*)$	c<1, $-K \le \theta \le \theta _b$
$$E^{**}$$	$$(U^{**}, I^{**}, V^{**})$$	c<1, $$\theta _a<\theta \le \theta _b$$

The stability of boundary equilibria are identical to the Gompertz case and summerised as the following theorem.

#### Theorem 3

(Boundary equilibria of the logistic model) For model (2),

(i) if c>1, then $E_1(K,0,0)$ is locally asymptotically stable, and
(ii) equilibrium $E_2(\theta ,0,0)$ is always unstable when it exists.

#### Remark 1

The limiting behaviour near the BRIS $E_0=(0,0,0)$ is discussed directly from the original logistic model (2) in Appendix B.3, which shows that for 0<θ<K (strong Allee), BRIS $E_0(0,0,0)$ is locally attractive. For $\theta \le 0$ (weak Allee), $E_0$ is unstable.

This result confirms that a strong Allee effect guarantees extinction for sufficiently small initial tumour burdens. This is a key therapeutic advantage which is not found in models where no Allee effect is considered, since those models are usually characterised by an unstable point in the origin for biologically sound parameter values. This is seen, for instance, in (Jenner et al. 2018).

We now introduce two new threshold parameters, $\theta _{c}$ and $\theta _{d}$, which emerge whilst considering the stability conditions for the positive equilibria. Their explicit expressions follow from an algebraic analysis presented in Appendix B.3. For completeness, we have

$$\begin{aligned} \theta _c&= \dfrac{ KU^*(2b+c-bc)+K \theta _a(5b+2c-2bc) }{ (2K-U^*)(2b+c-bc) }, \\ \theta _d&= \dfrac{ -B-\sqrt{B^2-4AC} }{ 2A } <0. \end{aligned}$$

Here $\theta _d$ is the negative root obtained from the quadratic polynomial $g(x)=Ax^{2}+Bx+C$, with coefficients

$$\begin{aligned} A&=a^2(U^*-2K)^2(2b+c-bc),\\ B&=a\left( b+c+aKU^*+2aK\theta _a\right) \left( U^*-2K\right) (2b+c-bc)\\&\quad +a^2\theta _c\left( U^*-2K\right) ^2(2b+c-bc) +2a^2c^2dK,\\ C&=-a^2c^2d\left[ (K+\theta _c)U^*+2K(\theta _a-\theta _c)\right] . \end{aligned}$$

These thresholds partition the parameter space into regimes where $$E^{*}$$ and $$E^{**}$$ may change stability. Finally, for coexistence equilibria, stability requires analogous conditions to Theorem 2, which are summarised in the following theorem.

#### Theorem 4

(Coexistence equilibria of the logistic model (2)) Assume c<1 and consider thresholds $\theta _c$, $\theta _d$, as defined previously. Then:

(i) If $-K \le \theta < \theta _b$, $E^*$ is stable when $\theta < \theta _c + \theta _d$.
(ii) If $$\theta _a < \theta \le \theta _b$$, $$E^{**}$$ is always unstable.

Figure 3 shows that a Hopf bifurcation, for the parameters considered in this example, occurs at $\theta _{HB} \approx 20.76$ (parameters: a=0.0001, b=0.01, c=0.06). This is a value slightly lower than the Gompertz threshold, meaning that logistic growth implies a transition to oscillatory dynamics at lower Allee thresholds, due to different early stages of growth. Table 4 contrasts the two models across key features. The Gompertz case exhibits a pseudo-extinction phenomenon under weak Allee effects, corresponding to the parameter regime -0.1<θ<0: in this range, the tumour experiences a negative Allee threshold, so that no positive critical density is required for survival. Biologically, θ<0 indicates a weak Allee effect: even very small populations of tumour cells can persist and eventually regrow, despite appearing to decline toward zero for short, limited times. Clinically, this behaviour aligns with the concept of minimal residual disease, in which tumour burden falls below the limits of detection without true eradication. The resulting apparent “spontaneous remission” thus reflects pseudo-extinction rather than genuine clearance and can lead to recurrence that is difficult to predict. This behavior is absent in the Logistic model, which always predicts either stable coexistence or complete clearance. In the next Section, we consider different initial burdens and viral characteristic to emphasise the above distinctions, using time-series calibrated to clinical parameters.

The above analysis also clarifies the distinct roles of the two growth laws considered in this study. In contrast to the Logistic model (2), the coexistence equilibria of the Gompertz model (1) are determined by the transcendental condition (3). This transcendental structure allows the Gompertz framework to generate richer equilibrium configurations, including multiple coexistence states. By contrast, the Logistic model leads to an algebraic equilibrium condition and hence to a simpler threshold structure. This structural difference helps explain why pseudo-extinction and richer multistability arise in the Gompertz simulations, whereas the Logistic model acts as a more tractable benchmark for early-stage tumour expansion and for identifying behaviours that are growth-law dependent.

**Table 4:** Comparative analysis of Gompertz versus Logistic frameworks under Allee effects

Feature	Gompertz model (1)	Logistic model (2)
Early-stage growth	Unrealistically rapid	Biologically realistic
Late-stage saturation	Accurate deceleration	Underestimates slowdown
Equilibrium multiplicity	Up to 3 coexistence states	At most 2 coexistence states
Pseudo-extinction (weak Allee)	Yes (for $\theta \approx 0^-$)	No
Hopf bifurcation threshold	$\theta _{HB} \approx 29.28$	$\theta _{HB} \approx 20.76$
Parameter sensitivity	High (multiple GH points)	Moderate (single GH point)

GH denotes a generalized Hopf bifurcation

## Typical Time-Series, Model Comparison and Clinical Scenarios

We now explore theoretical predictions through numerical integration in clinically relevant parameter ranges, with baseline values taken from Kim et al. (2011) and Jenner et al. (2018). Following Jenner et al., the densities of tumour cells are scaled by $$\bar{U}=10^{6}$$ cells   $$\hbox {mm}^{-3}$$, so that U=1 corresponds to $$10^{6}$$ cells   $$\hbox {mm}^{-3}$$; time is measured in days. All parameter definitions and units are summarised in Table 1, together with the baseline values used in the simulations. Specifically, we take m=0.01, ξ=0.01, γ=0.06 and K=100 for the Gompertz model (1); for the logistic model (2) we use a=0.0001, b=0.01 and c=0.06. To relate the Gompertz growth parameter *m* to clinically reported doubling times, note that colorectal tumours typically exhibit a doubling time ($$T_d=12 $$ ) of approximately 10–15 days during the exponential phase (Jenner et al. 2018). At U≈K/e (the maximum growth point for Gompertzian dynamics), this yields $$m \approx \ln 2/[T_d\ln (K/U)]\approx 0.058$$, indicating that the baseline choice m=0.01 represents a slower-growing tumour with a doubling time of about 70 days. For the logistic model  (2) this implies the relation a≈m/K=0.0001.

To make the narrative of this section explicit before the individual figures are discussed, Table 5 summarises the main regimes, the corresponding figures and their principal dynamical and therapeutic messages.

**Table 5:** Summary of representative dynamical regimes analysed in Sect. 4

Regime	Figures	Main message
High viral clearance	Fig. 4	Threshold-separated basins under rapid viral clearance.
Weak or marginal Allee regimes	Fig. 5	Multiple stable coexistence or near-zero pseudo-extinction in Gompertz model.
Hopf-near transients	Figs. 6, 11	Long oscillatory transients and sensitivity to initial conditions.
Comparisons	Figs. 7, 8, 9 and 10	Different early-growth rates, Hopf structures and parameter analysis in Gompertz and Logistic models.

### Gompertz Model: Extinction, Coexistence and Pseudo-Extinction

**Fig. 4:**
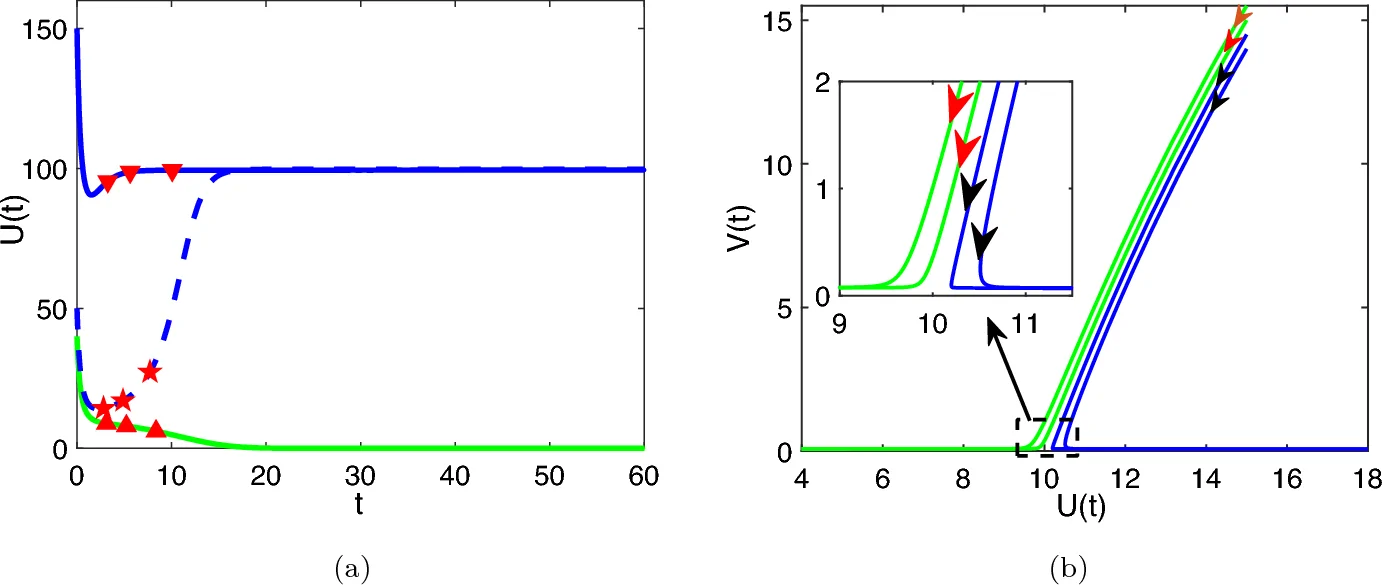
Typical, selected trajectories that describe the stability behaviour of model (1) for the parameter choice γ=1.5>1 and θ=10. Panel (a) shows selected trajectories of *U*(*t*), illustrating threshold-separated outcomes under rapid viral clearance. Panel (b) shows the corresponding phase portrait near the fixed threshold θ=10, with arrows indicating the direction of the solution curves. In all panels, blue and green curves represent trajectories that ultimately converge to $\overline{E}_1$ and $\overline{E}_0$, respectively. Time is in days (Color figure online)

Figure 4a shows examples of rapid viral clearance, for a high value of γ=1.5 (half-life 0.67 days). The strong Allee threshold θ=10 highlights bistable responses: trajectories of high initial tumour loads ($U_0 > 50$) converge to carrying capacity $\overline{E}_1(100,0,0)$, while low initial loads ($U_0 < 10$) approach extinction $\overline{E}_0$. The value of θ=10 represents the boundary of the basin that separates different regimes. Note also how some tumour populations transiently decay but then spike within 20 days, consistent with some clinical observations showing that viruses can become quickly undetectable and can elicit tumours regrowth within 48 h, despite high initial titers (Zhong et al. 2025).

Figure 4b explores threshold proximity effects near θ=10, looking at the sensitivity of trajectories close to θ=10. Starting from $U_0$ slightly above that value and with varying viral loads, we observe sharp extinction transitions: tumours below threshold collapse within 15–20 days, while those remaining above threshold “rebound” to $\overline{E}_1$. Importantly, it appears that under oncolytic viruses the threshold is slightly shifted and, for these parameters, increased by about one per cent of its original value. Therapy can modestly enlarge the extinction basin and, with this choice of θ, allow initial tumours to grow approximately $10^5$ cells per $\text {mm}^3$ over the threshold but still be eradicated. These values are small but clinically not completely irrelevant.

Usually, it is known that high clearance rates (γ>1) preclude durable, positive responses, even at large dosing values. This has led to techniques to make viral vectors more successful, by engineering persistence in the samples. Some experimental examples, with an appropriate and recent mathematical model, can be found in Pooladvand and Kim (2022).

**Fig. 5:**
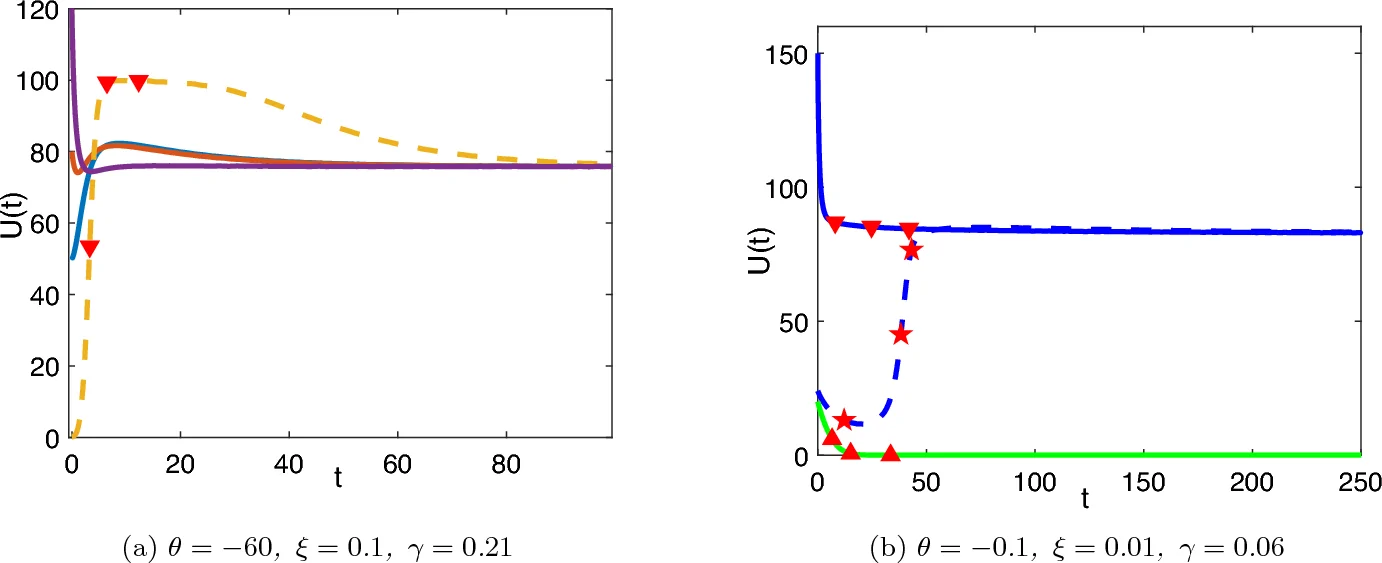
Weak Allee outcomes in the Gompertz model (1). Panel **a** corresponds to θ=-60, ξ=0.1, and γ=0.21, where all displayed trajectories converge to an ordinary stable coexistence state. Panel **b** corresponds to the marginal weak Allee regime θ=-0.1, ξ=0.01, and γ=0.06, where three coexistence equilibria exist: $$\overline{E}^{*},\,\overline{E}^{**},\,\overline{E}^{***}$$. $$\overline{E}^{*}$$ is stable, $$\overline{E}^{**}$$ is unstable (separating basins of attraction), and $$\overline{E}^{***}$$ is a near-extinction equilibrium with magnitude numerically negligible and indistinguishable from the extinction state $$\overline{E}_0$$, which is a distinctive feature of the Gompertz model (1) (Color figure online)

Two strong Allee cases are not repeated as a separate time-series panel here. First, in the unconditional-extinction regime with $\theta =40 > \theta _{LP} \approx 29.33$: Its equilibrium structure follows from Fig. 2, where $\theta _{LP}$ denotes a saddle-node (noted as a “limit point” by the software) bifurcation. This parameter region represents maximal therapeutic leverage: tumours are intrinsically non-viable below a high threshold, and viral pressure ensures populations never escape the extinction basin. Clinically, this scenario models an aggressive, first approach that reduces tumour density below a critical level, after which residual disease self-eliminates via Allee suppression.

Second, the therapeutically critical strong Allee case θ=10, γ=0.06: As indicated by the equilibrium branches in Fig. 2, three equilibria coexist: stable $\overline{E}^*=(61.4, 961.6, 160.3)$, unstable $\overline{E}_2(10,0,0)$, and attractive $\overline{E}_0$. This case follows the same threshold-separation principle highlighted in Fig. 4, but now in a lower-clearance regime where coexistence is also dynamically possible. We next turn to the weak-Allee regimes, where the Gompertz model displays ordinary coexistence and, near marginal negative thresholds, pseudo-extinction.

Figure 5a explores weak Allee effects (θ=-60, ξ=0.1, γ=0.21) yielding unique stable coexistence. All trajectories converge to $\overline{E}^*=(58.2, 235.8, 39.3)$ within 80 days. This represents chronic infection where viral replication balances tumour growth, producing a quiescent equilibrium. Panel (a) shows smooth convergence without oscillations, indicating strong negative feedback ($h > z_2$). Such states can be therapeutically advantageous, to some extent, if the tumour burden can be minimised to harmless levels and then disease can be controlled without increasing the toxicity of the treatment to dangerous values. However, sustained viral pressure is required: this is an ideal scenario and implies that treatment cessation can risk relapse and escape from this balance between virus and cancer.

At low clearance values, the Gompertzian case displays a unique and non trivial feature: Fig. 5b illustrates what we call a “pseudo-extinction”, under marginal weak Allee effects (θ=-0.1). Three equilibria exist, with one basically zero: $$\overline{E}^* \approx (82.8, 1296.8, 216.1)$$, an unstable $$\overline{E}^{**}\approx (5.2, 81.4, 13.6)$$ and $$\overline{E}^{***} \approx (0,0,0)$$. Panel (b) shows trajectories showing bistability: a high-density coexistence regime between virus and tumour, and an effectively extinct population.

Tumours reduced below a critical density (e.g., in this case U<1) may transition from a coexistence state to pseudo-extinction. As a representative example, fixing all other parameters and choosing $\theta =-0.2, \gamma = 0.21$, yields a pseudo-extinction equilibrium at $U \approx 6.78 \times 10^{-7}$ (or $U \approx 9.04 \times 10^{-4}$ when $\theta =-0.2, \gamma = 0.3$ ). Biologically, a larger viral clearance rate γ reflects faster elimination of free virus, which reduces sustained oncolytic pressure and shifts the pseudo-extinction equilibrium to a higher, yet still low, residual tumour level. This mechanism mimics spontaneous remissions when residual tumour has a size below detectability, as observed in clinical practice. Indeed, the logistic case does not produce this behavior, but only allows either stable coexistence or true extinction.

All this shows that the Allee term could provide good therapeutic implications: pushing the cancer below the threshold U<θ implies a full eradication outcome. It remains to be seen how this can be accomplished through existing techniques beyond virotherapy. For example, the use of checkpoint inhibitors, genetic ablation or immune boosting may be useful to alter the tumour microenvironment and enhance the Allee effect.

**Fig. 6:**
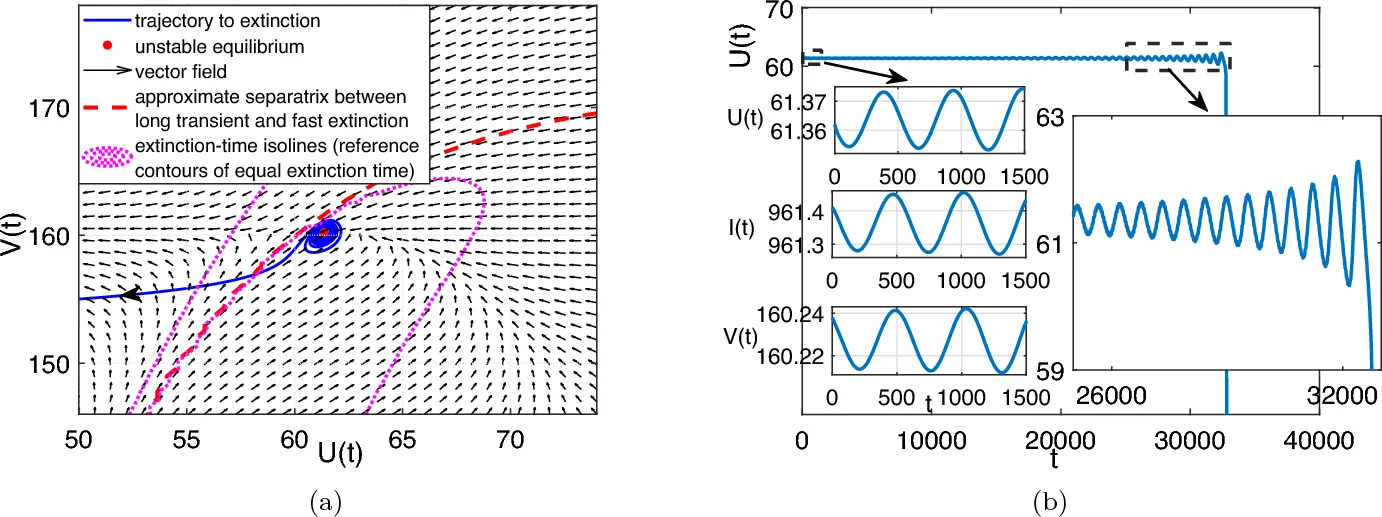
Oscillatory, bistable dynamics above the Hopf threshold for ξ=0.01, γ=0.06, and $\theta = 29.2830 > \theta _{HB}$. Panel **a** shows the (*U*, *V*) slice at $I = I_{0}=961.1$, including the unstable coexistence equilibrium (red point), the local vector field (black arrows), and extinction-time isoclines. The red dashed curve approximates a slow–fast separatrix: initial conditions inside it (lower right) generate prolonged oscillations before extinction, whereas those outside (upper left) decay rapidly. Panel **b** presents the corresponding temporal evolution, where trajectories spiral away from $\overline{E}^{*}$ and undergo long-lived oscillatory transients prior to collapse. The inset highlights the slower viral dynamics: viral peaks *V*(*t*) increase incrementally each cycle while tumour troughs *U*(*t*) and *I*(*t*) gradually decline until crossing a critical threshold that then triggers a rapid extinction (Color figure online)

### Hopf-Near Dynamics and Slow–Fast Separatrices

Near the Hopf point in Fig 2, $\theta _{\textrm{HB}}\approx 29.28$, the system exhibits two distinct oscillatory behaviours. For slightly subcritical values (e.g. $\theta = 29.2798 < \theta _{\textrm{HB}}$), trajectories exhibit small-amplitude damped oscillations that decay monotonically toward the coexistence equilibrium $\overline{E}^{*}$. Such decay is consistent with the equilibrium remaining a stable focus prior to the (subcritical) Hopf bifurcation, and therefore no separate figure is shown. By contrast, Fig. 6$(\theta = 29.2830 > \theta _{\textrm{HB}})$ shows the behaviour that a slight increase beyond the Hopf threshold produces: oscillations that persist for tens of thousands of days before a sudden drop to extinction. These very long simulated transients should not be interpreted as realistic clinical waiting times, but their relevance is diagnostic: they identify parameter regimes in which early trajectories remain close to a slow basin boundary, so that small changes in initial tumour burden or viral load can determine whether clearance is fast or delayed. Panel (a) presents the (*U*, *V*) phase portrait on the slice $I = I_{0} = 961.1$, including the unstable coexistence equilibrium (red point), the local vector field, and extinction-time isoclines. The red dashed curve acts as a slow–fast separatrix: initial conditions on its lower-right undergo prolonged oscillatory transients, whereas those on the upper-left decay rapidly toward extinction. Panel (b) displays the associated temporal dynamics. Oscillation amplitudes grow gradually over approximately $3.3\times 10^{4}$ days as viral peaks *V*(*t*) rise and tumour troughs values *U*(*t*) and *I*(*t*) subsequently decline further. Once *U*(*t*) enters a narrow critical window (around U≈55), extinction occurs within roughly 30 days. Even under identical parameter values, initial states with small differences in initial tumour burden or viral load may have very different, long term outcomes.

Clinically, it is difficult to obtain actionable indications from this scenario, but the message seems to be that an apparently stable disease trajectory may ultimately collapse to extinction, whereas premature cessation of therapy may leave the system trapped within the slow-oscillation basin. This calls for extra care when dealing with long-term strategies of tumour management or recurrent, chronic conditions such as some types of leukemias and myeloproliferative diseases, which are known to be affected by persistent oscillations in tumour burdens (Chikkappa et al. 1980; Xiao et al. 2003).

**Fig. 7:**
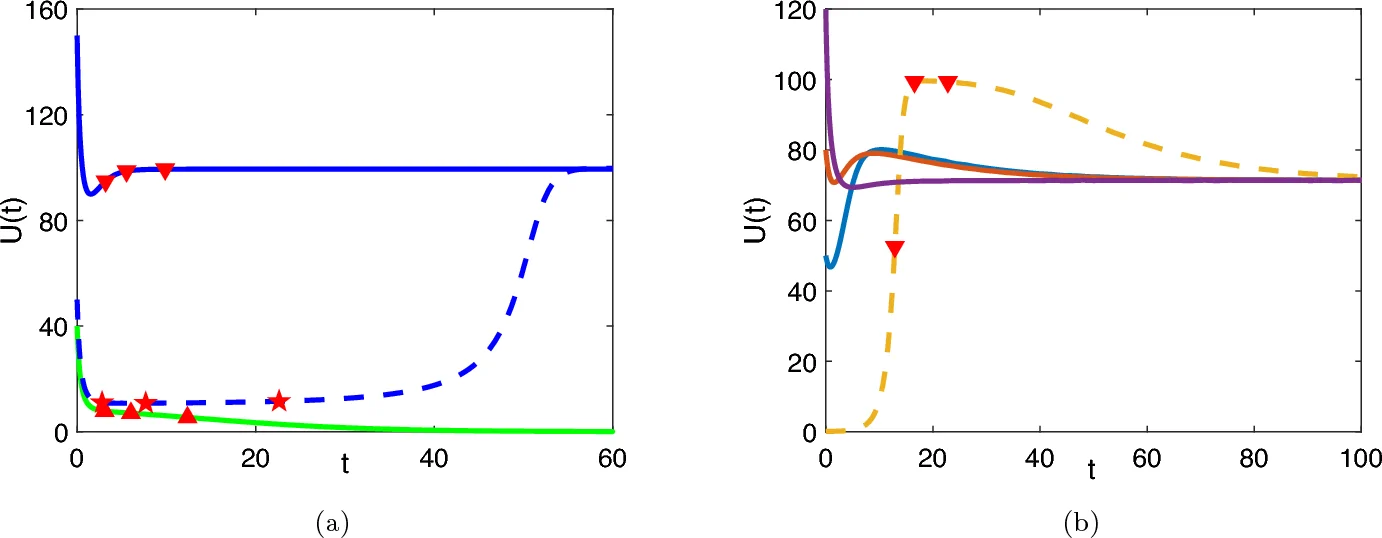
Dynamics and stability of outcomes of model (2) under different Allee and clearance regimes. Panel **a** corresponds to θ=10 with large viral clearance rate (c=1.5), and panel **b** to θ=-60 with small clearance rate (c=0.21). Trajectories are shown for different initial tumour densities and are compared with the corresponding results in Figs. 4a and 5a of model (1) (Color figure online)

### Comparison between Gompertz and Logistic Models

Let us now look at the logistic growth model and discuss it in the light of the findings for Gompertz dynamics. Figures 4, 5 and 6 reveal broadly similar stability patterns in the Gompertz framework, but key quantitative differences persist when the logistic model is used as a benchmark. Comparing Fig. 7a with Fig. 4a and Fig. 7b with Fig. 5a, there appear early-stage growth discrepancies between the Gompertz and logistic models under different parameter regimes and initial tumour densities. For the parameter set with strong Allee effect and large clearance rate, $(\theta =10, \gamma =c=1.5)$, shown in Figs. 4a (Gompertz model) and 7a (Logistic model), the two models exhibit noticeable differences at low initial tumour density (e.g. $U_0 = 5$). Notwithstanding the presence of viral pressure, the Gompertz trajectories still display a faster initial rise and approach the coexistence equilibrium earlier than the logistic case. This reflects the stronger effective early-growth rate induced by the Gompertz law at low densities, whereas the logistic model is characterised by a moderate, density-limited initial growth. Instead, for high initial density (e.g. $U_0 = 95$), both models converge to equilibrium within a similar time window, and the discrepancy in early growth becomes less pronounced, since the dynamics are rapidly dominated by saturation near the carrying capacity.

Furthermore, Figs. 5a (Gompertz model) and 7b (logistic model) correspond to the weak Allee effect and low clearance rate $(\theta =-60, \gamma =c=0.21)$. In that case, the presence of a negative Allee parameter and reduced clearance rate alter the early interaction between tumour and virus. At low initial tumour density, the Gompertz model still reaches its equilibrium faster than the logistic model, but the difference in convergence time is smaller than in the high clearance case, as viral dynamics more strongly modulate the effective growth. For high initial density, the trajectories of the two models are again very close, with both solutions relaxing to the equilibrium on comparable time scales.

Overall, these differences are important indicators of how tumours dissimilarly react to virotherapy: the early growth discrepancies between Gompertz and logistic models are most evident at low tumour burden and are amplified in the high clearance regime $$(\theta =10, \gamma =c=1.5)$$, whereas they are partially masked by viral feedback in the weak Allee effect, low clearance regime $$(\theta =-60, \gamma =c=0.21)$$. One important difference is also that the logistic model lacks multistability under marginal weak Allee effects. At θ=-0.1, only two equilibria exist, i.e. a stable $$E^*$$ and an unstable $$E^{**}$$, contrasting with the Gompertz model’s three (including $$\overline{E}^{***}$$). This topological simplicity eliminates pseudo-extinction as a therapeutic outcome, meaning that a logistic growth under weak Allee effects either terminates in a detectable coexistence or in a complete clearance, with no intermediate quasi-extinct states.

Close to the Hopf point, the logistic model, reported in Appendix C (Fig. 11), exhibits a phase-space organisation that is qualitatively similar to the Gompertz case. In both cases, trajectories spiral outwards from the unstable coexistence equilibrium $E^*$, whilst extinction-time isoclines delineate a slow–fast separatrix that partitions the phase space into two distinct regions: one supporting long-lived oscillatory transients and another characterised by rapid extinction. However, in the logistic model the separatrix has a sharper curvature and the region sustaining prolonged oscillations is visibly narrower. This gives rise to a heightened sensitivity to initial conditions in the vicinity of the Allee threshold, which might have some clinical repercussions. In Fig. 11 (b) the representative trajectory undergoes sustained oscillations for approximately $1.7 \times 10^4$ days before collapsing to extinction, a shorter transient than that observed in the logistic model(Fig. 11(b)). This reduction in transient lifetime is consistent with the geometric structure revealed in panel (a): when the slow region is compressed, trajectories positioned nearer the fast side of the separatrix reach extinction more rapidly. Consequently, the disparity in extinction times between the logistic and Gompertz models is more naturally attributed to the reorganisation of the slow–fast geometry than to qualitative changes in the underlying oscillatory mechanism.

Taken together, Figs. 6 and 11 demonstrate that oscillatory dynamics above the Hopf threshold are governed by a common mechanism: long-lived oscillatory transients emerge near a slow–fast separatrix across which trajectories undergo abrupt collapse to extinction. In both cases, trajectories initialised in proximity to this boundary may exhibit quasi-stable oscillations over very long model times before transitioning abruptly to extinction. The clinically relevant implication is the sensitivity to initial tumour burden and viral load, rather than the actual simulated duration of the oscillations.

From a clinical perspective, this sensitivity suggests that modest differences in initial conditions or early treatment response may influence qualitatively distinct long-term outcomes, even under identical model parameters, or impact the management of long-term cronic conditions.

### **Sensitivity Analysis**

We are now interested in systematically mapping how viral parameters ξ and γ modulate bifurcation thresholds, identifying possible therapeutic windows where modest dose adjustments yield significant improvements to outcomes.

**Fig. 8:**
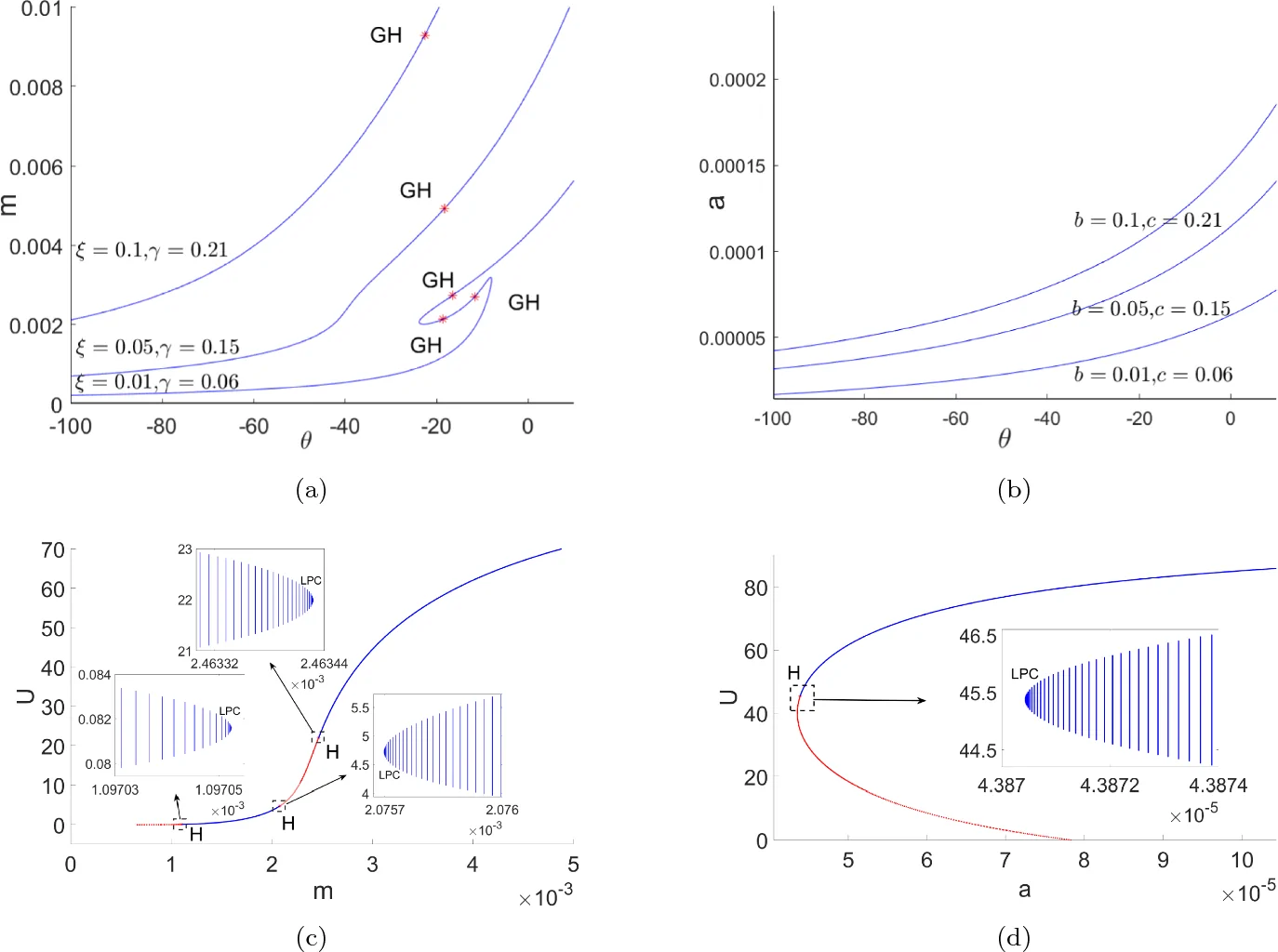
Comparison of Hopf bifurcation structures in the Gompertz (**a**), **c** and logistic (**b**), **d** models under varying parameters. Panels (**a**)–(**b**) show two-parameter continuations of Hopf bifurcations in the (m,θ) and (a,θ) plane for different values of $(\xi ,\gamma )$ and (*b*, *c*). **a** Gompertz case: GH points occur throughout, with branches switching from supercritical (upper-right) to subcritical (lower-left). **b** Logistic case: branches remain subcritical across all θ. Panels **c**–**d** have θ=-20, ξ=b=0.01 and γ=c=0.06, and examine one-parameter bifurcations in *m* and *a*. **c** Gompertz case: three Hopf points emerge along the equilibrium branch and give rise to alternating stable and unstable limit cycles. **d** Logistic case instead reveals a single subcritical Hopf point under identical parameters (Color figure online)

Figure 8, obtained using the software MATCONT (Dhooge et al. 2003), plots the loci of Hopf points in (m,θ) space for three pairs of parameters $(\xi , \gamma )$, looking at Gompertz (panels (a) and (c)) and logistic (panels (b) and (d)) growths. In these codimension-two curves, note that Generalized Hopf (GH) points mark transitions between supercritical and subcritical points. Panel (a) reveals remarkable sensitivity in the Gompertz model at low ξ=0.01, γ=0.06 (bottom curve). Three GH points emerge, creating an S-shaped curve where stability alternates: as θ decreases (from right to left) we encounter an unstable branch that turns into stable and finally unstable again. This non trivial structure implies that reducing the Allee threshold through therapeutic intervention can induce multiple regime shifts. For intermediate parameters (ξ=0.05, γ=0.15, middle curve), only one single GH persists and the curve remains smooth, whereas at high parameters (ξ=0.1, γ=0.21, top curve), the GH point slides rightward and the curve becomes nearly linear. On the other hand, panel (b) shows that the logistic case have loci that remain subcritical across all θ’s, regardless of $(\xi , \gamma )$. This is a reflection of the model’s simpler topology: coexistence equilibria always destabilise into growing oscillations that drive extinction, never showing damped oscillations. Hence, tumours that are less likely to exhibit prolonged oscillatory coexistence directly go from stable equilibrium to extinction. This could be an interesting idea to investigate experimentally.

Figure 8c–d now focuses on a direct comparison of codimension-one continuation diagrams, concentrating on Hopf bifurcation structures in the Gompertz and logistic models. Parameter values are identical, e.g. θ=-20, ξ=b=0.01, and γ=c=0.06, with tumour growth rate *m*(*a*) varied as the bifurcation parameter. Blue branches indicate stable equilibria, red indicate unstable. As *m* increases from 0.001 to 0.003, the system undergoes stability transitions: stable coexistence, then oscillations, then “renewed” coexistence and finally oscillations again, associated with three Hopf points along the equilibrium branch. The three insets show representative limit cycles emerging at each Hopf point; their stability (supercritical versus subcritical) is determined by the sign of the first Lyapunov coefficient and is consistent with the branch colouring and the presence/absence of nearby unstable cycles.

Notably, the switching criticality across Hopf points reflects underlying structural bifurcations (e.g., GH points), consistent with transitions traced in panel (a). Panel (d) shows the logistic counterpart, where only a single Hopf point arises, subcritical in nature, with no reversal in direction: the Gompertz case allows richer oscillatory bifurcation patterns, including direction switching and multi-stage transitions, while the logistic model remains dynamically simpler. This sensitivity suggests that some tumours with intrinsically high, Gompertz-like growth rates near Hopf thresholds are clinically less predictable.

**Fig. 9:**
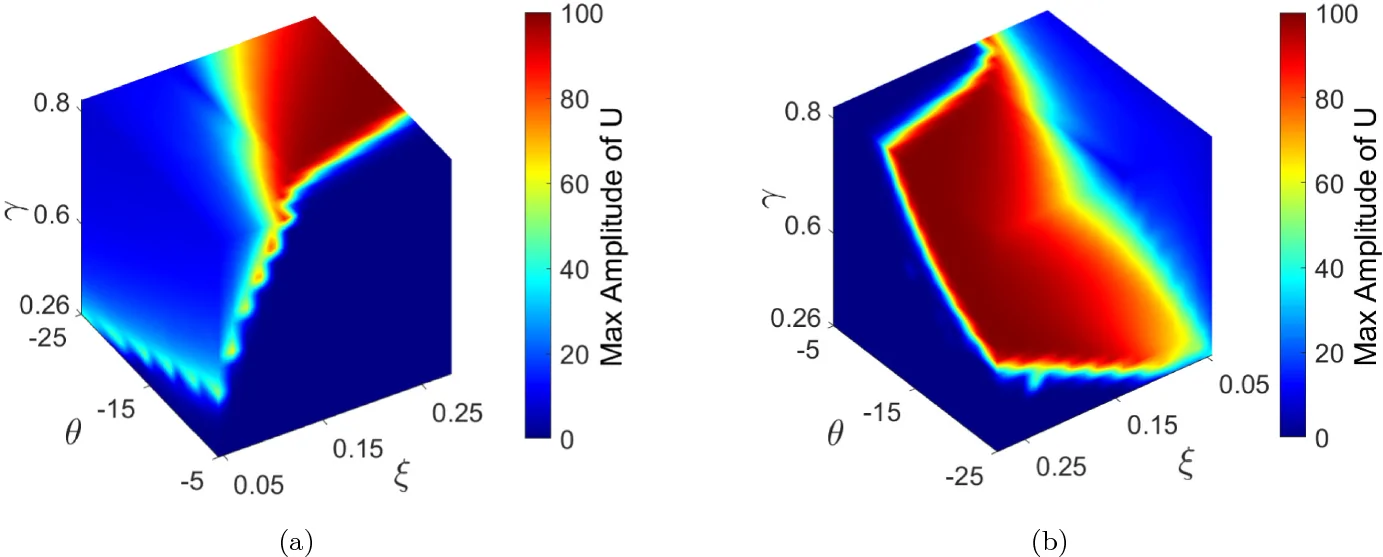
Amplitude of stable limit cycles in the Gompertz growth version of model (1) under varying parameter conditions, with fixed m=0.0021. Panels **a** and **b** display two perspectives of the $(\theta ,\xi ,\gamma )$ parameter space, where colour indicates the maximal amplitude of *U* along the stable limit cycle (warmer tones correspond to larger amplitudes) (Color figure online)

**Fig. 10:**
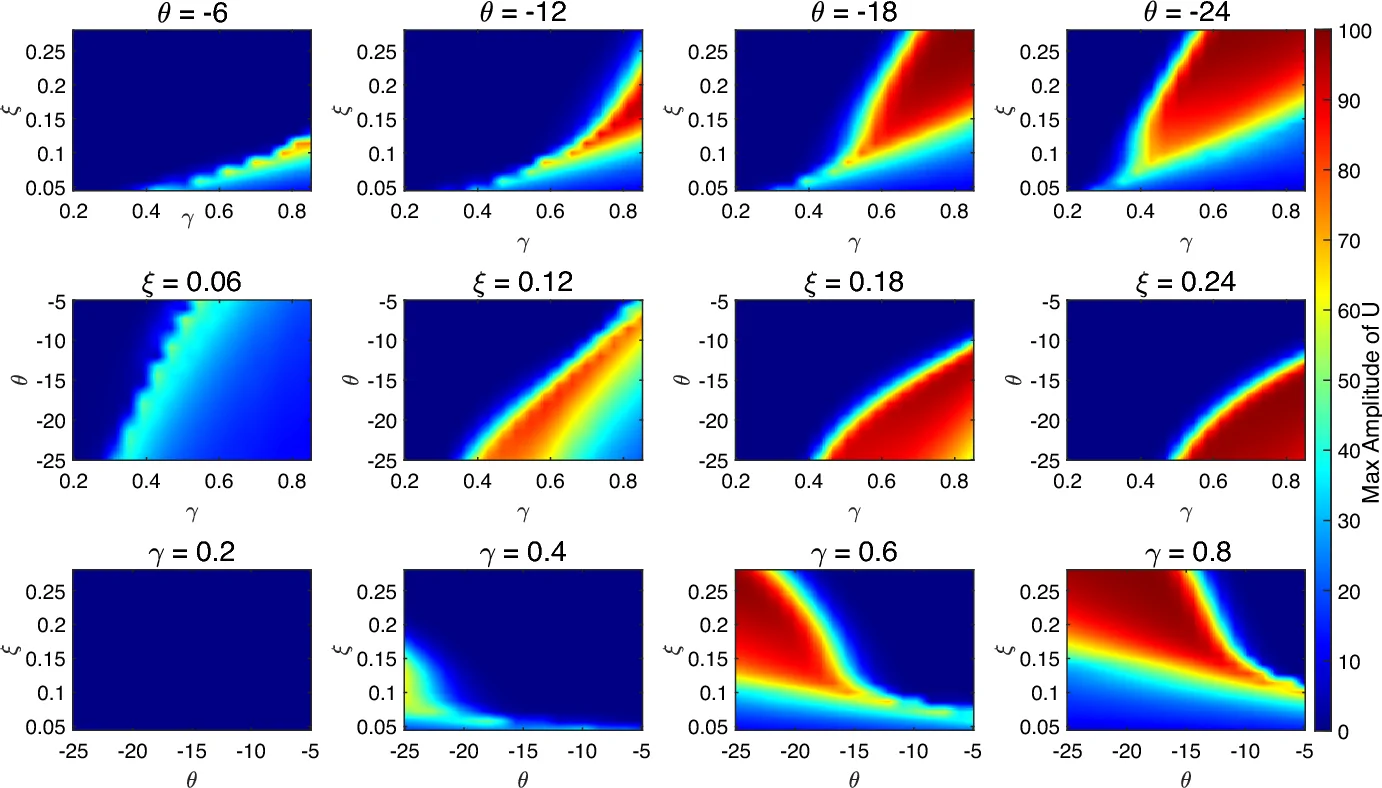
Dependence of max amplitude of tumour cell density *U* on model parameters θ (Allee threshold), ξ (lysis rate), and γ (viral clearance rate) in the Gompertz growth version of model (1). Results are shown in three sets of two-parameter projections. High-amplitude regimes (red) correspond to large excursions in tumour values, low-amplitude regions (blue) represent suppressed or almost eradicated tumours (Color figure online)

Variation in the amplitude of tumour burden when oscillatory behaviour takes place can obviously have important therapeutic consequences. Figures 9 and 10 show how the Allee threshold θ, tumour lysis rate ξ, and viral clearance rate γ jointly affect the amplitude of stable limit cycles in model (1), under a fixed tumour growth rate m=0.0021. Colours indicate the maximum *U* amplitude along the cycle, with red being the highest and blue the lowest. The two three-dimensional views in Fig. 9 reveal a wedge-shaped high-amplitude region embedded in the $(\theta ,\xi ,\gamma )$ cube. Large oscillations occur only within this narrow wedge, while the surrounding parameter space corresponds to low-amplitude oscillations or non-oscillatory dynamics. This suggests that sustained oscillations arise from a coordinated balance between tumour cells co-operation (encoded by θ) and viral kinetics ($\xi ,\gamma$), rather than from extreme values of any single parameter.

Figure 10 provides two-parameter “slices” that indicate how the high-amplitude area in Fig. 9 projects into $(\xi ,\gamma )$-planes at fixed θ (top row), into $(\theta ,\gamma )$-planes at fixed ξ (middle row), and finally $(\theta ,\xi )$-planes at fixed γ (bottom row). For weak Allee effect (e.g. $\theta \in [-24,-6]$), increasing both the lysis rate ξ and the clearance rate γ drives the system sharply from a low-amplitude regime (blue) into a high-amplitude regime (yellow-red), where a narrow band describes the transition. The appearance of these structures is directly related to the complexity in how Hopf points distribute in the space of parameters $(\theta ,\xi ,\gamma )$, also suggesting that any direct approach to alter the Allee threshold (for example CNDP2 inhibition (Guzelsoy et al. 2025)) might negatively impact the therapy, for example driving the system from areas of lower amplitude to areas of higher ones.

In fact, also “slices” at fixed ξ further reveal a non-monotone dependence of amplitude on viral clearance rate γ. When γ is very small, free virus persists for long periods and continually suppresses tumour regrowth; trajectories then converge to a coexistence state with only modest fluctuations in *U*, and the system remains in a low-amplitude (blue) regime. At the opposite extreme, when γ is very large, virus particles die almost immediately after lysis, severely limiting viral replication but increasing the inflow of new viral particles that replace the dead ones, so that tumour dynamics overall stays close to a low-amplitude regime. Between these extremes there exists an intermediate-to-high clearance window in which lysis generates appreciable viral bursts, while clearance is still slow enough to permit transient viral amplification. This alternation between short phases of viral expansion and subsequent rapid decay produces a controlled “boom-bust” pattern in tumour load, corresponding to the narrow yellow-red bands in Fig. 10.

From a clinical standpoint, such moderate oscillations may offer a therapeutic window. If the virus is able to sensibly reduce the burden without inducing large increases in the tumour volume, it could promote clonal selection or malignant progression, then controlled oscillations might be a form of possible management for particular tumours, for example haematological ones. For instance, these types of conditions are known to have significant oscillations in white and red blood cell numbers that still can be managed successfully throughout the life of the patients  (Kennedy 1970; Chikkappa et al. 1980; Rodriguez and Lutcher 1976). Further, these plots could be useful for treating cases where tumours show small, recurrent oscillations (i.e., they reside in the “blue zone” of the plots): it could well be the case that those tumours may be completely eradicated if virotherapy is integrated with other therapies for example immune boosting or checkpoint blockades, that can definitely and completely drive the tumour to full extinction.

## Discussion

The use of mathematical models incorporating Allee effects into oncolytic virotherapy frameworks shows that treatment outcomes can be fundamentally altered and hints at new possibilities in therapeutic strategies. Three central findings have emerged from our analysis: bistability under strong Allee effects enables threshold-based eradication; pseudo-extinction under weak Allee effects can be a source of spontaneous remissions and parameter sensitivity near Hopf bifurcations can guide better dosing strategies.

The Allee effect in cancer has been underexplored despite some experimental evidence. For example, cooperative nutrient scavenging can be seen as a density-dependent mechanism satisfying formal Allee effect criteria in Guzelsoy et al. (2025). More broadly, several experimentally grounded mechanisms can generate Allee-type growth limitations in tumour cell populations, including feedback mediated by autocrine signalling (Gerlee et al. 2022) and cooperative interactions between clones via diffusible metabolites (Hershey et al. 2023). Such effects have also been reported in glioblastoma contexts relevant to post-resection recurrence, where growth at low density is markedly impaired in a manner consistent with an Allee threshold (Neufeld et al. 2017).

The models that we discuss incorporate this biological term using the parameter θ, quantifying the minimally viable tumour density below which cooperative mechanisms fail. Positive θ’s (strong Allee effect) imply the existence of an extinction threshold, which is sensitive to proper clinical strategies; negative θ’s (weak Allee effect) indicates cooperative deficits at low density without an absolute threshold, which are relevant in establishing the value of the minimal residual tumour allowed by the model. This is shown, for example, in (Jin et al. 2021).

Additional mechanisms supporting tumour Allee effects also include, for instance, collective immune evasion, where small populations cannot saturate cytotoxic lymphocytes (Dunn et al. 2002), paracrine signalling, when growth factors reach concentrations that are ineffective (Delitala and Ferraro 2020) and mechanical cooperation, where insufficient cell-cell contacts hinder proliferations due to a lack of correct signalling (Johnson et al. 2019). The relative contributions of these mechanisms likely vary across cancer types, explaining part of the typical heterogeneity in observed thresholds. Our framework accommodates this diversity in a simple, direct way by considering θ as a measurable patient-specific parameter rather than a universal constant.

Figure 4a, together with the equilibrium branches in Fig. 2, demonstrates that strong Allee effects (θ>0) create bistable systems where initial tumour burden determines the ultimate fate. Strategies that pursue a burden U<θ guarantee extinction regardless of subsequent viral dynamics, while cases when U>θ point to tumour-virus coexistence. Nothwithstanding the simplicity of the models, these outcomes provide some general ideas about possible strategies. For example, a two-phase treatment protocol where, firstly, the clinician pursues the reduction of tumour density below threshold using surgical resection (or other aggressive techniques) and then administers oncolytic virus to accelerate clearance of residual cells seems to be a viable, promising option, coherent with our findings. We show that dosing can be moderate since the presence of Allee effects ensure extinction, with the virus merely shortening the descent of cell numbers towards zero (Nguyen et al. 2022a; Alwithenani et al. 2025; Du et al. 2025). Note also that there is evidence of some success of neoadjuvant checkpoint blockade prior to virotherapy (Ribas et al. 2017), which can be in part attributed to Allee effects too.

An interesting finding of this paper is in Fig. 5b, which reveals that marginal weak Allee effects (-1<θ<0) produce pseudo-extinction states where tumours persist at vanishingly low densities (fewer than one cell per $mm^3$). This phenomenon, present only for the Gompertz case, is in line with observations of minimal residual disease that enter prolonged dormancy before eventual clearance (Cañellas-Socias et al. 2022).

Estimating effective patient-specific values of θ is intrinsically difficult, because θ is an effective threshold summarising low-density cooperative mechanisms rather than a directly measurable anatomical quantity. A first feasible route is model-based inference from longitudinal low-burden or post-resection recurrence data: Neufeld et al. (2017) used glioblastoma recurrence dynamics to support weak Allee-type growth, showing that clinical growth histories can constrain an effective threshold parameter. A second route is to use experimentally calibrated low-density growth assays to set priors on whether θ should be positive, marginally negative, or far below zero. (Johnson et al. 2019) showed that cancer-cell population growth at low densities can deviate from exponential growth and is better described by Allee-type models in controlled in vitro assays. (Gerlee et al. 2022) further showed that autocrine signalling can generate both weak and strong Allee effects, with consistency against patient-derived brain-tumour cell-line data. These approaches would not identify θ uniquely for every patient, but they could provide plausible ranges for risk stratification and for testing whether a tumour is near an Allee-sensitive regime.

**Fig. 11:**
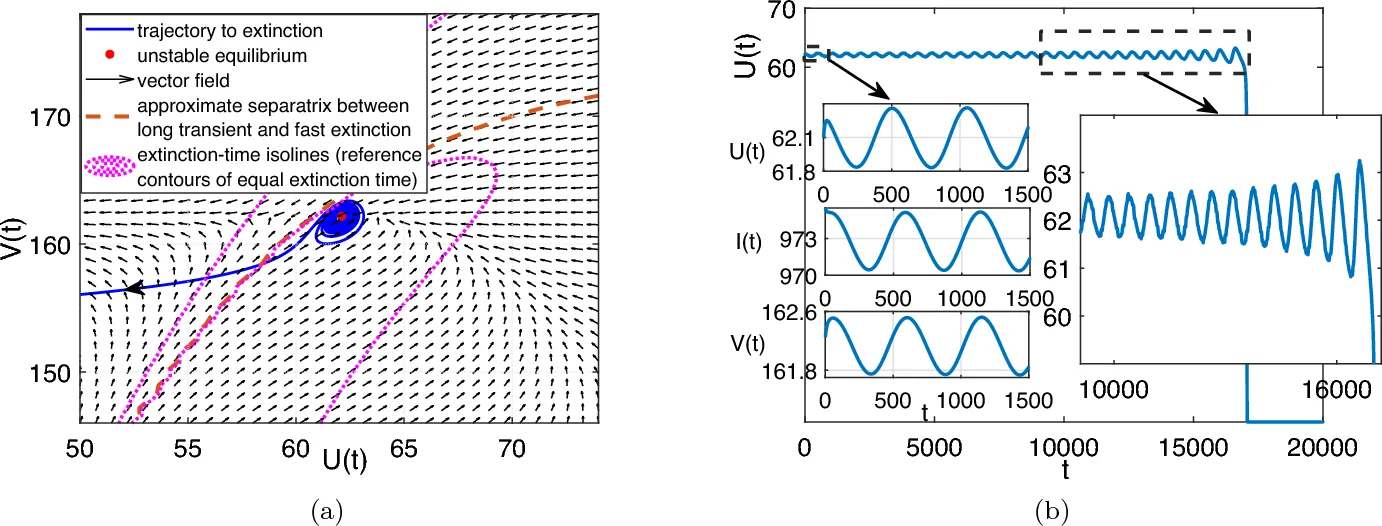
Additional Logistic Hopf-near transient used as a supplementary benchmark comparison. Oscillatory, spiral trajectories above the Hopf threshold for the logistic model (2), highlighting differences from the Gompertz growth case. Here b=0.01, c=0.06, and $\theta =20.7628>\theta _{\textrm{HB}}$. Panel **a** shows the (*U*, *V*) slice at $I=I_0=972.9$, including the unstable coexistence equilibrium $E^*$ (red point), the local vector field (black arrows), and extinction-time isoclines. The red dashed curve approximates a slow–fast separatrix, separating initial conditions that generate long-lived oscillatory transients (lower right) from those leading to rapid extinction (upper left). Panel **b** presents the corresponding temporal evolution, where trajectories spiral away from $E^*$ and undergo extended oscillatory transients prior to collapse (Color figure online)

Figure 6 and Figs. 9 and 10 reveal that tumours with parameters near Hopf thresholds exhibit prolonged oscillations before extinction, and the corresponding Logistic benchmark is reported in Appendix C (Fig. 11). The magnitude of these oscillations amounts to thousands or tens of thousands of cells. These very long transients (tens of thousands of days in silico) pose a dilemma if translated into clinical practice. It is never safe to leave tumours unattended but it could be worse to continue invasive therapies for too long, especially if tumour oscillations appear to be controlled. Our separatrix analysis (Figs. 6a) provides some helpful quantitative guidance. The red dashed curves delineate fast versus slow clearance basins in (*U*, *V*) phase space. Measuring tumour density and viral load during early cycles (first 100 days) allows to have a projection onto this plane, that can predict whether extinction will occur within months or years. Patients in the slow basin are candidates for intensification of therapy: increasing viral dose (an upward shift in phase space) or adding cytoreductive agents (a leftward shift) accelerates clearance.

Solutions initially close to this boundary may display quasi-stable oscillations over clinically relevant timescales, yet eventually rapidly transition to extinction once they drift across the separatrix. Thus, small perturbations in initial tumour burden or viral load can produce qualitatively different long-term outcomes under identical parameters. This is a key point and reinforces the idea of early intervention being often critical.

Within this framework, the two-parameter slices in Fig. 10 further reveal how the Allee threshold, lysis rate, and clearance rate combine to determine whether this oscillatory window widens or collapses. Large-amplitude oscillations (yellow-red regions) arise only within a narrow wedge of $(\theta ,\xi ,\gamma )$ values where tumour depletion, regrowth, and viral cycles all contribute to the dynamics. Outside this wedge, either Allee suppression or tumour clearance disrupts the cycle, leading to small oscillations or monotone decay. Large oscillations are undesirable as they may promote clonal selection and often correspond to malignant progression. The blue regions in Figs. 9 and 10 therefore identify more favourable regimes in which oscillations are damped or absent. Treatment strategies should aim to shift the system away from high-amplitude zones, particularly when early dynamics place trajectories close to the separatrix.

Alternatively, extended oscillations themselves may be exploitable (Hastings et al. 2018) when minima occur and residual cells are maximally vulnerable to complementary therapies. Synchronising chemotherapy pulses with minima in oscillations could achieve eradication. This “oscillation-guided adaptive dosing” represents a novel paradigm still in its infancy, where mathematical models directly dictate treatment schedules. All this is far from being implemented efficiently in the clinic but it clearly has potential.

Table 4, Figs. 4, 5 and 6, Fig. 7, and Appendix C (Fig. 11) provide a direct comparison between the Gompertz model (1) and Logistic model (2). The two models share the same biological interpretation of the Allee term, but they differ in how growth slows near the carrying capacity and in how low-density dynamics interact with viral infection. The Gompertz model is more appropriate in capturing late-stage saturation and thus exhibits richer multistability, including pseudo-extinction. The logistic model provides a different dynamics for early growth and has different long-term, late-stage behaviours, lacking pseudo-extinction. Thus, the Logistic model is useful as a comparative reference, whereas the Gompertz framework is better suited for interpreting delayed clearance, marginal persistence and recurrence-like low-density outcomes. Such different models can be suited to different clinical cases, providing clinicians with choices based on individual patient details.

Despite our models’ insights, their mathematical formulation is such that several biological complexities that may modulate Allee thresholds and treatment outcomes are absent. It is important to clearly highlight these, so that the reader is aware of the limitations that our models carry. For example, CD8+ T cells, Natural Killer (NK) cells and macrophages exhibit nonlinear density-dependent activation (Su et al. 2023). At low tumour densities, antigen presentation may be insufficient to sustain effector populations, effectively raising the Allee threshold. Conversely, checkpoint inhibitors could lower θ by enhancing immune activity at low density. Immune pathways (Li et al. 2024) are absent in our model, and their inclusion would likely modify several quantitative conclusions. For example, immune-mediated viral clearance would increase the effective clearance rate and could therefore shrink, or even eliminate, coexistence regimes that require sustained viral pressure. At the same time, immune cytotoxicity or checkpoint-mediated activation could alter the effective Allee threshold by changing tumour survival at low density. We therefore expect the threshold-based mechanism to persist qualitatively whenever density-dependent tumour growth remains present, but the exact locations of equilibria, Hopf thresholds and coexistence regimes might need to be re-assessed and may be altered in an immune-extended model.

Furthermore, a well-mixed ODE framework ignores spatial heterogeneity in both tumour and viral distributions. Allee effects in spatial models produce more complex dynamics than the one discussed here, for example traveling wave solutions where invasion fronts stall below critical densities (Grumbach and Hilker 2025). Spatial formulations could identify spatially localised extinction strategies that are absent in our approach. Also, we only consider tumours as completely solid, ignoring the possibility of necrotic cores and angiogenesis, which can and do affect tumours even at the sizes we model in this work (Nishida et al. 2006; Liu and Jiao 2019). These important aspects in our view deserve focussed approaches in the future.

Probably one of the most important limitation is that there are no viral injections in our approach. Rather, viral particles are always ready to be elicited, with a continuous inflow of viral loads. This gives rise to lengthy, prolonged coexistence (Fig. 5b). Besides this scenario not being entirely realistic, it is important to bear in mind that tumours tend to evolve and develop resistance to infection, making the efficiency of virotherapy decreasing with time (Moorman et al. 2025). Also, there are experiments that show glutamine-dependent cooperation, with tumours utilising diverse metabolic strategies and responses (Guzelsoy et al. 2025). All this means that tumours may exhibit qualitatively different Allee thresholds due to different nutrient diffusion and absorption.

Despite these limitations, our framework provides some immediate, straightforward guidelines to experimentalists. Also, bistability maps (Figs. 2, 3) and parameter sensitivity analyses (Figs. 8, 9 and 10) give some general advice about therapy design. An eligibility criteria for specific therapies could exclude patients with estimated $h < z_2$ (high oscillation risk) from single-agent virotherapy, instead enrolling them in combination regimens. How to determine θ or $z_2$ (or similar measures) is clearly a challenge.

Finally, let us remark that one of the main results of this work is to show oncolytic virotherapy outcomes are fundamentally affected by density-dependent tumour dynamics encapsulated in Allee effects. Exploiting Allee thresholds through strategic sequencing of surgery, invasive therapies and virotherapy could highly reduce required viral loads while lowering recurrence rates. Measuring and incorporating Allee effects into cancer treatment schedules has the potential to make virotherapy a more effective, better complement to existing strategies. Future work integrating immune dynamics, spatial structures, tumour heterogeneity, vascular dynamics and angiogenesis, immunoediting and resistance to virus infection is one of the challenges ahead, carrying the promise of better outcomes for a variety of tumours.

## Data Availability

Data will be made available on request.

## Other Possible Models with Inferior Biological Realism

This Appendix discusses other model choices that differ from the two discussed in Sect. 2 and evaluate four alternatives, analysing their respective strengths and weaknesses (Table 6).

**Table 6:** Comparative evaluation of Allee factor functional forms, including the models discussed in the main text

Property	Model 5	Model 6	Model 7	Model 8	Model 1
Strong Allee capable	Yes	No	No	Yes	Yes
Weak Allee capable	No	Yes	Yes	No	Yes
Bounded at high *U*	No	Yes	Yes	No	Yes
Smooth near U=0	No	Yes	Yes	Yes	Yes
Parameter flexibility	Low	Low	Low	Low	High
Biological realism	Moderate	Moderate	Moderate	Low	High

**Logarithmic Strong Allee Gompertz Model:**

5 $$\begin{aligned} {\left\{ \begin{array}{ll} \dfrac{dU}{dt} = & mU \ln \left( \dfrac{K}{U} \right) \ln \left( \dfrac{U}{\theta } \right) - \dfrac{UV}{U+I},\\ \dfrac{dI}{dt} = & \dfrac{UV}{U+I} - \xi I,\\ \dfrac{dV}{dt} = & \xi I -\gamma V. \end{array}\right. } \end{aligned}$$

As per previous cases, *U*, *I*, *V* still represent uninfected tumour cells, infected tumour cells, and free virus; *K* is the carrying capacity and $\Omega (U,\theta )=\ln \left( \dfrac{U}{\theta }\right)$, $\theta \in (0, K)$ defines the Allee threshold; *m*, ξ, γ are growth, lysis, and clearance rates (Table 1) (Amarti et al. 2018). The model (5) has these properties: Ω<0 for U<θ (negative growth); Ω=0 at U=θ (threshold); Ω>0 for U>θ (positive growth). Thus, very small tumours decline rapidly, whereas above threshold growth is positive but decelerates as U→K, which is a canonical strong Allee profile. Cooperative benefits grow logarithmically with density, however, the net growth rate goes to -∞ as $U \rightarrow 0^+$, producing an unrealistic collapse dynamics.

**Saturating Weak Allee Gompertz Model:**

6 $$\begin{aligned} {\left\{ \begin{array}{ll} \dfrac{dU}{dt} = & mU \ln \left( \dfrac{K}{U} \right) \dfrac{U}{U+\theta } - \dfrac{UV}{U+I},\\ \dfrac{dI}{dt} = & \dfrac{UV}{U+I} - \xi I,\\ \dfrac{dV}{dt} = & \xi I -\gamma V, \end{array}\right. } \end{aligned}$$

where $\Omega (U,\theta )=\dfrac{U}{U+\theta }$ with θ>0 (Courchamp et al. 2008; Zu et al. 2010; Cushing 2015). Its properties show that $\Omega \in (0,1)$ for all U>0 (always positive); $\Omega \rightarrow 0$ as U→0 (severe suppression at low density); $\Omega \rightarrow 1$ as $U \rightarrow \infty$ (saturates to baseline growth). This captures a weak Allee effect: growth efficiency is impaired at low density but rises monotonically toward the baseline Gompertz rate $m\ln \left( \dfrac{K}{U}\right)$, with θ acting as a half-saturation constant. Note that there is no extinction threshold (weak Allee only) and threshold θ must be positive.

**Exponential Weak Allee Gompertz Model:**

7 $$\begin{aligned} {\left\{ \begin{array}{ll} \dfrac{dU}{dt} = & mU \ln \left( \dfrac{K}{U} \right) \left( 1-e^{- U/\theta } \right) - \dfrac{UV}{U+I},\\ \dfrac{dI}{dt} = & \dfrac{UV}{U+I} - \xi I,\\ \dfrac{dV}{dt} = & \xi I -\gamma V, \end{array}\right. } \end{aligned}$$

where $\Omega (U,\theta )=1-e^{- U/ \theta }$ with θ>0 (Dennis 1989; Courchamp et al. 2008). Here $\Omega (U,\theta )$ represents an effective fraction of tumour cells (or growth opportunities) that experience sufficient cooperation/interaction to contribute to net growth at burden *U*, and properties are highly similar to the previous model  (6). For small *U*, $\Omega (U,\theta )\approx U/\theta$, hence growth is strongly reduced; as *U* increases, $\Omega (U,\theta )\rightarrow 1$, and the model reduces to the standard Gompertz term. The parameter θ is the saturation scale (larger θ means slower saturation).

**Linear Strong Allee Gompertz Model:**

8 $$\begin{aligned} {\left\{ \begin{array}{ll} \dfrac{dU}{dt} = & \bar{m} U \ln \left( \dfrac{K}{U} \right) \left( \dfrac{U}{\theta }-1 \right) - \dfrac{UV}{U+I},\\ \dfrac{dI}{dt} = & \dfrac{UV}{U+I} - \xi I,\\ \dfrac{dV}{dt} = & \xi I -\gamma V, \end{array}\right. } \end{aligned}$$

where $\Omega (U, \theta )={U}/{\theta }-1$ is the most commonly used mathematical form for describing the Allee effect (Courchamp et al. 2008; Fadai and Simpson 2020). Its properties show that Ω<0 for U<θ (negative growth); Ω=0 at U=θ (threshold); Ω>0 for U>θ (positive growth); $\Omega \rightarrow +\infty$ as $U \rightarrow +\infty$ (unbounded). This means that linear dependence on density implies cooperation benefits grow proportionally with population size. But a critical flaw exists here too: unbounded corrections at high density violate biological realism and overestimate cooperation in dense populations, where instead competition dominates.

Although all models above bear some interest and have some positive aspects that capture part of the dynamics of the Allee terms, only the two presented in the main text are worth considering in details and be used for bases for insights that contain some clinical value.

## Mathematical Derivations

This appendix contains the details and derivations for the technical proofs of theorems stated in Sect. 3.

### Equilibrium Classification of Model (1)

**Case 1: Weak Allee effect (** θ<0 **).** Let us define

9 $$\begin{aligned} f(x) = m(x-\theta ) \ln \left( \frac{K}{x} \right) , \quad x>0. \end{aligned}$$

Differentiating *f*(*x*) with respect to *x* yields:

10 $$\begin{aligned} f'(x) = m \left[ \ln \left( \frac{K}{x} \right) - 1 + \frac{\theta }{x} \right] . \end{aligned}$$

The second derivative is given by $$f''(x) = -\dfrac{m}{x^2}(x+\theta )$$. We observe that $f^{\prime }\left(K\right)=m\left(\theta /K-1\right)<0$. The function $f^{\prime }\left(x\right)$ is strictly increasing on (0,-θ) and strictly decreasing on $$(-\theta , +\infty )$$, achieving its global maximum at x=-θ. Thus, the maximum value is

$$ f'(-\theta ) = m \left( \ln \left( \dfrac{K}{-\theta } \right) - 2 \right) . $$

If $$f'(-\theta ) \le 0$$, corresponding to $$\theta \le -K e^{-2}$$, then $f^{\prime }\left(x\right)\leq 0$ for all x>0. Consequently, *f*(*x*) is monotonically decreasing. Given f(K)=0 and $$\lim _{x \rightarrow 0^+} f(x) = +\infty $$, there exists a unique solution $$\overline{U_1}^*$$ to the equation $$f(x) = \xi (1- \gamma )/\gamma $$. Specifically, the unique $$\overline{U_1}^*$$ satisfies

$$ \overline{U_1}^* \exp \left\{ \frac{\xi (1-\gamma )}{m \gamma (\overline{U_1}^*-\theta )} \right\} = K, \quad \text {if } \theta \le -K e^{-2}. $$

Conversely, if $f^{\prime }\left(-\theta \right)>0$, implying $$\theta > -K e^{-2}$$, the equation $f^{\prime }\left(x\right)=0$ has two different roots, denoted by $$x_1$$ and $$x_2$$ (with $$x_1< -\theta < x_2$$). The function *f*(*x*) is decreasing on $$(0, x_1)$$, increasing on $$(x_1, x_2)$$, and decreasing on $$(x_2, +\infty )$$. The roots of $f^{\prime }\left(x\right)=0$ satisfy $$x e^{-\theta /x} = K/e$$. Let $$z=-\dfrac{\theta }{x}$$, we have $$-z e^{-z}=\dfrac{\theta }{K}e$$. By using the Lambert *W* function, we have $$z=-W\left( \dfrac{\theta }{K}e\right) $$ and $$x=\dfrac{\theta }{W\left( \dfrac{\theta }{K}e\right) }$$. Then we obtain:

11 $$\begin{aligned} x_1 = \dfrac{\theta }{W_{-1}\left( \dfrac{\theta e}{K} \right) } \quad \text {and} \quad x_2 = \dfrac{\theta }{W_0\left( \dfrac{\theta e}{K} \right) }. \end{aligned}$$

Let $\Lambda = \dfrac{\xi (1- \gamma )}{\gamma }$. The existence of roots for Eq. (3) depends on Λ:

- If $\Lambda \in (0, f(x_1)) \cup (f(x_2), +\infty )$, Eq. (3) has a unique root.
- If $\Lambda \in \{ f(x_1), f(x_2) \}$, Eq. (3) has two different roots.
- If $\Lambda \in (f(x_1), f(x_2))$, Eq. (3) has three different roots.

**Case 2: **θ=0. In this scenario, Eq. (3) simplifies to $mU \ln (K/U) = \Lambda$. Defining f(x)=mxln(K/x), we have:

$$ f'(x) = m \left( \ln \left( \frac{K}{x} \right) - 1 \right) , $$

and $f^{\prime \prime }\left(x\right)=-m/x<0$, $f^{\prime }\left(K\right)=-m<0$, $$\underset{x \rightarrow 0^+}{\lim }f'(x)=+ \infty $$. Hence, $f^{\prime }\left(x\right)$ is strictly decreasing, with a unique root at $$x_3 = K/e$$. Since f(K)=0 and $$\lim _{x \rightarrow 0^+} f(x) = 0$$, *f*(*x*) increases on $$(0, x_3)$$ and decreases on $$(x_3, K)$$, achieving a maximum $$f(x_3) = mK/e$$. Depending on the value of Λ:

- If Λ>mK/e, Eq. (3) has no roots.
- If Λ=mK/e, there are two equal roots.
- If Λ<mK/e, there are two different roots.

Note that as $\theta \rightarrow 0^-$, $x_2 \rightarrow K/e = x_3$, consistent with the limit.

**Case 3: Strong Allee effect** (θ>0). From Eq. (10), we have $f^{\prime \prime }\left(x\right)<0$ for all x>0. With $$\lim _{x \rightarrow 0^+} f'(x) = +\infty $$ and $f^{\prime }\left(K\right)<0$, $f^{\prime }\left(x\right)$ is strictly decreasing. There exists a unique critical point $$x_4 \in (0, K)$$ such that $$f'(x_4)=0$$. Solving for this point yields:

$$ x_4 = \dfrac{\theta }{W_0 \left( \dfrac{\theta e}{K} \right) }. $$

Since θ>0, the argument $\dfrac{\theta e}{K}$ is positive, so only the principal real branch $W_0$ is defined (the branch $W_{-1}$ is real-valued only for arguments in [-1/e,0)). Therefore, $x_2=\dfrac{\theta }{W_0 \left( \frac{\theta e}{K}\right) },$ and the critical point previously denoted by $x_4$ coincides with $x_2$ in this regime (the $W_{-1}$-branch point $x_1$ does not exist for θ>0). Moreover, *f*(*x*) is strictly increasing on $(0,x_2)$ and strictly decreasing on $(x_2,+\infty )$.

Also, we have f(θ)=0, f(K)=0 and *f*(*x*) has its maximum when $x=x_2$, that is, $f(x_2)=f(\frac{\theta }{W(\frac{\theta }{K}e)})$. We also find $f(x_2)=f(\frac{\theta }{W(\frac{\theta }{K}e)})\rightarrow f(x_3)=\frac{mK}{e}$ if $\theta \rightarrow 0^+$. Depending on the value of Λ when θ>0:

- If $\Lambda > f(x_2)$, Eq. (3) has no roots.
- If $\Lambda = f(x_2)$, there are two equal roots.
- If $\Lambda < f(x_2)$, there are two different roots.

As $\theta \rightarrow 0^+$, the critical point $\underset{\theta \rightarrow 0^+}{\lim }x_2 = K/e= x_3$. Moreover, the value of *f*(*x*) at these points likewise converges: $\underset{\theta \rightarrow 0^+}{\lim }f(x_2) = mK/e = f(x_3)$. For clarity and consistency across regimes, we adopt a unified notation $x_2=\dfrac{\theta }{W(\frac{\theta }{K}e)}$ for the critical point where *f*(*x*) achieves its local maximum (previously denoted as $x_3$, or $x_2$ depending on θ), applicable for all $\theta \in [0, K)$.

### Gompertz Model Stability (Theorems 1–2)

The Jacobian matrix of model (1) evaluated at equilibrium (*U*, *I*, *V*) is:

12 $$\begin{aligned} \begin{aligned} J&= \begin{pmatrix} \partial _U f_1 & \partial _I f_1 & \partial _V f_1 \\ \partial _U f_2 & \partial _I f_2 & \partial _V f_2 \\ 0 & \xi & -\gamma \end{pmatrix}\\&= \left( \begin{matrix} m(2U-\theta ) \ln \left( \frac{K}{U}\right) -m(U-\theta )-\frac{VI}{(U+I)^2} & \frac{UV}{(U+I)^2} & -\frac{U}{U+I} \\ \frac{VI}{(U+I)^2} & -\xi -\frac{UV}{(U+I)^2} & \frac{U}{U+I} \\ 0 & \xi & -\gamma \\ \end{matrix} \right) . \end{aligned} \end{aligned}$$

where $f_1, f_2, f_3$ denote the right-hand sides of the three equations in (1). The characteristic polynomial is given by:

13 $$\begin{aligned} \begin{aligned}&\lambda ^3 + \lambda ^2 \Bigl [ \gamma + \xi + m(U - \theta ) - m(2U - \theta )\ln \left( \frac{K}{U}\right) + \frac{V}{U+I} \Bigr ] \\&+ \lambda \Bigl [ \gamma \xi + \left( m(U - \theta ) - m(2U - \theta )\ln \left( \frac{K}{U}\right) \right) \left( \gamma + \xi + \frac{UV}{(U+I)^{2}}\right) \\&\qquad + \gamma \frac{UV}{(U+I)^{2}} + (\gamma + \xi )\frac{VI}{(U+I)^{2}} - \frac{U}{U+I}\xi \Bigr ] \\&+ \left( m(U - \theta ) - m(2U - \theta )\ln \left( \frac{K}{U}\right) \right) \left( \gamma \xi + \gamma \frac{UV}{(U+I)^{2}} - \frac{U}{U+I}\xi \right) \\ &\quad + \gamma \xi \frac{VI}{(U+I)^{2}} = 0. \end{aligned} \end{aligned}$$

**Proof of Theorem **1: At $\overline{E}_1(K,0,0)$, the characteristic polynomial factorises as $(\lambda - m(\theta -K))(\lambda ^2 + (\xi +\gamma )\lambda + \xi (\gamma -1)) = 0$. The first root $\lambda _1 = m(\theta -K) < 0$ for θ<K. The quadratic has discriminant $\Delta = (\xi -\gamma )^2 + 4\xi > 0$, yielding two real roots $\lambda _{2,3} = [-(\xi +\gamma ) \pm \sqrt{\Delta }]/2$. For γ>1, both $\lambda _2, \lambda _3 < 0$, establishing stability. At $\overline{E}_2(\theta ,0,0)$, the first root becomes $\lambda _1 = m\theta \ln (K/\theta ) > 0$ for 0<θ<K, proving instability. □

**Proof of Theorem **2: At coexistence equilibrium $\overline{E}^*$, substituting $I^* = [(1-\gamma )/\gamma ]U^*$ and $V^* = [\xi (1-\gamma )/\gamma ^2]U^*$ yields characteristic polynomial $\lambda ^3 + a_2\lambda ^2 + a_1\lambda + a_0 = 0$ with coefficients:

$$\begin{aligned} a_2&= \xi + \gamma + h, \\ a_1&= h(\xi + \gamma + \xi (1-\gamma )) - \xi ^2(1-\gamma )/\gamma , \\ a_0&= \xi \gamma h(1-\gamma ), \end{aligned}$$

where $$h = m(U^*-\theta ) - mU^*\ln (K/U^*)$$. Routh-Hurwitz conditions require: (i) $$a_0> 0 \Rightarrow h > 0$$, (ii) $$a_2 > 0$$ (always satisfied if h>0), (iii) $$a_1 a_2 - a_0 > 0$$. Expanding (iii) and solving the resulting quadratic inequality in *h* yields $$h > z_2$$, where $$z_2$$ is given by Eq. (14) below. For equilibrium $$\overline{E}^{**}$$, the condition $$U^{**} < (K+\theta )/2$$ implies $$f'(U^{**}) > 0$$, hence h<0 and $$a_0 < 0$$, proving instability. Equilibrium $$\overline{E}^{***}$$ lies in the decreasing region of *f*, yielding h>0 and implying stability when $$h > z_2$$. □

Note that the expression for $z_2$ is given by the following:

14 $$\begin{aligned} \begin{aligned} z_2&=\frac{-\Bigl ((\xi +\gamma )(2\xi +\gamma -\xi \gamma ) -\frac{\xi ^2(1-\gamma )}{\gamma }-\xi \gamma (1-\gamma )\Bigr )}{2(2\xi +\gamma -\xi \gamma )} \\&+\frac{\sqrt{\Bigl ((\xi +\gamma )(2\xi +\gamma -\xi \gamma ) -\frac{\xi ^2(1-\gamma )}{\gamma }-\xi \gamma (1-\gamma )\Bigr )^2 +4(2\xi +\gamma -\xi \gamma )(\xi +\gamma )\frac{\xi ^2(1-\gamma )}{\gamma }}}{2(2\xi +\gamma -\xi \gamma )}. \end{aligned} \end{aligned}$$

### Logistic Model Stability (Theorems 3–4)

Proofs follow a structure that is very similar to the Gompertz case.

Proof of Theorem 3: At $E_1(K,0,0)$, the characteristic polynomial is $(\lambda - aK(\theta -K))(\lambda ^2 + (b+c)\lambda + b(c-1)) = 0$. Stability requires c>1, yielding all negative eigenvalues. At $E_2(\theta ,0,0)$, the first root becomes $\lambda _1 = a\theta K^2 > 0$ for 0<θ<K, proving instability. □

Proof of Theorem 4: The stability analysis for the logistic case again follows the same passages as in the Gompertz case. Most algebraic steps carry over verbatim once the substitution $I^* = [(1-c)/c]U^*$, $V^* = [b(1-c)/c^2]U^*$ and $h \mapsto e = -aU^{*}(K+\theta -2U^{*})$ is applied, and the resulting characteristic coefficients $(a_2,a_1,a_0)$ retain an analogous dependence on the equilibrium $U^{*}$. To avoid repetition, we do not reproduce the parts of the derivation that are identical to the previous analysis. Instead, we present only the different terms in $-K\le \theta \le \theta _a$. The expressions below therefore isolate the components that are unique to the logistic model case.

We have the following. First:

$$\begin{aligned} \theta _c=\frac{KU^*(2b+c-bc)+K \theta _a(5b+2c-2bc)}{(2K-U^*)(2b+c-bc)}, \end{aligned}$$

then,

$$\begin{aligned}&a_1 a_2-a_0 \\ &=a^2(U^*-2 K)^2(2 b+c-b c)(\theta -\theta _c)^2 \\ &\quad +[a(b+c+a K U^*+2 a K \theta _a)(U^*-2 K)(2 b+c-b c) \\ &\quad +a^2 \theta _c(U^*-2 K)^2(2 b+c-b c) +2 a^2 c^2 d K -a^2c^2dU^*](\theta -\theta _c) \\ &\quad - a^2 c^2 d[(K+\theta _c)U^*+2K(\theta _a-\theta _c)]. \end{aligned}$$

Let

15 $$\begin{aligned} g(x)&= a^2(U^*-2 K)^2(2 b+c-b c){x}^2 - a^2 c^2 d[(K+\theta _c)U^*+2K(\theta _a-\theta _c)] \nonumber \\ &+[a\left( b+c+a K U^*+2 a K \theta _a\right) \left( U^*-2 K\right) (2 b+c-b c) \nonumber \\ &+a^2 \theta _c\left( U^*-2 K\right) ^2(2 b+c-bc) +2 a^2 c^2 d K]~x. \end{aligned}$$

Then we have $a^2(U^*-2 K)^2(2 b+c-b c)>0$ and x<0. If x=0, we get $g(0)=- a^2 c^2 d[(K+\theta _c)U^*+2K(\theta _c-\theta _a)]=- a c^2 de<0$. Thus, there must exist $x=\theta _d<0$ ($x=\theta _d$ is the negative root of equation g(x)=0) such that $g(\theta _d)=0$, that is, g(x)>0 for $x<\theta _d$. The expression of $\theta _d=\frac{-B-\sqrt{B^2-4 A C}}{2 A}<0$, and $A=a^2(U^*-2 K)^2(2 b+c-b c)$, $B=a\left( b+c+a K U^*+2 a K \theta _a\right) \left( U^*-2 K\right) (2 b+c-b c)+a^2 \theta _c\left( U^*-2 K\right) ^2(2 b+c-b c)+2 a^2 c^2 d K$, and $C=- a^2 c^2 d[(K+\theta _c)U^*+2K(\theta _a-\theta _c)]$.

Thus, in the regime $-K\le \theta <\theta _a$, the model admits a unique positive equilibrium $E^*$. Since this regime already guarantees $\theta <\theta _a$, the Routh–Hurwitz conditions reduce to the additional requirement $\theta <\theta _c+\theta _d$. Hence, $E^*$ is locally asymptotically stable if $-K\le \theta <\theta _a$ and $\theta <\theta _c+\theta _d$.

The case $$\theta _a<\theta <\theta _b$$ can be treated in the same way. In this regime, there exist two positive equilibria $$E^*$$ and $$E^{**}$$. The lower-branch equilibrium $$E^{**}$$ is always unstable, whereas the upper-branch equilibrium $$E^*$$ is locally asymptotically stable if $$\theta <\theta _c+\theta _d$$.

In conclusion, for the case of $-K\le \theta < \theta _b$, if c<1 and $\theta < \theta _c+\theta _d$, the positive equilibrium $E^*$ is locally asymptotically stable.

**BRIS Stability (Remark** 1 **):**

Although the BRIS $E_0=(0,0,0)$ of the logistic model (2) does not mathematically exist, it is biologically relevant so we now discuss the model dynamical behaviour in a small neighbourhood of it, particularly for U≪1. In fact, we can, in what follows, show that in the biologically relevant positive region, the BRIS $E_0$ is locally attractive under strong Allee effect (namely, 0<θ<K) and it is unstable under weak Allee effect (namely, $-K\le \theta \le 0$).

#### Under Strong Allee effects, that is When 0<θ<K.

Using the fact that U=0 is an invariant plane of the system, the original system reduces to

16 $$\begin{aligned} \frac{dI}{dt}=-bI,\qquad \frac{dV}{dt}=bI-cV. \end{aligned}$$

Consider the trajectory with any initial value $(0,I_0,V_0)$ where $I_0>0$ and $V_0>0$. Thus, as $t\rightarrow \infty$, we obtain that $I(t)=I_0e^{-bt}\rightarrow 0$ and $V(t)=(bI_0t+V_0)e^{-ct}\rightarrow 0$ for b=c or $V(t)=\frac{bI_0}{c-b}e^{-bt}+\left( V_0-\frac{bI_0}{c-b}\right) e^{-ct}\rightarrow 0$ for b≠c. Thus, any trajectory initiated from the invariant plane will not only remain on the plane but also approach $E_0$ as $t\rightarrow \infty .$

Next, we consider the case where a trajectory initiated from $(U_0,I_0,V_0)$ with $0<U_0\ll 1.$ Then, there exists a constant $0<\delta <\min \{\theta ,K\}$ such that $0< U_0<\delta <\theta$. From the first equation of model (2), we obtain $0\le U(t)<\delta <\theta$ for all t≥0. Denote $\eta =a(K-\delta )(\theta -\delta ).$ Then, η>0 and using the first equation again we obtain that

$$ \frac{dU}{dt} < -\eta U, $$

implying U(t)→0 as $t\rightarrow \infty$. We now use the result from the first case or (16), and obtain that all trajectories initiated from $(U_0,I_0,V_0)$ with $0<U_0\ll 1$ will approach $E_0$ as $t\rightarrow \infty .$

Therefore, when 0<θ<K, the BRIS $E_0$ is locally attractive.

#### Under Weak Allee Effects, that is When $-K\le \theta \le 0$.

Under the weak Allee effect, we can see that trajectories initiated from the invariant line I=V=0 will move away from the $E_0$ when $U_0$ is small. In fact, any such trajectory will remain on the invariant line, because U(t)>0,I(t)=V(t)=0 for all t≥0. Then, the first equation becomes

$$ \frac{dU}{dt}=aU(K-U)(U-\theta ), $$

which implies that U(t)→K as $t\rightarrow \infty .$ Thus, the BRIS is unstable under the weak Allee effect.

## Additional Logistic Hopf-Near Transient

A Figure similar to Fig. 6 is also obtained for the Logistic case.
